# Ultrathin and ultrastrong hydrogel bioelectronic membranes

**DOI:** 10.1093/nsr/nwag105

**Published:** 2026-02-12

**Authors:** Mingze Sun, He Zhang, Xingdao He, Xi Wei, Binbin Cui, Hao Huang, Hao Li, Yuan Lin, Shiming Zhang, Zhong Alan Li, Peng Shi, Lizhi Xu

**Affiliations:** Department of Mechanical Engineering, The University of Hong Kong, Hong Kong 999077, China; Department of Cell Biology, Third Military Medical University, Chongqing 400038, China; Department of Mechanical Engineering, The University of Hong Kong, Hong Kong 999077, China; Advanced Biomedical Instrumentation Centre Limited, Hong Kong 999077, China; Department of Biomedical Engineering, City University of Hong Kong, Hong Kong 999077, China; School of Medicine, Wuhan University of Science and Technology, Wuhan 430081, China; Department of Electrical and Electronic Engineering, The University of Hong Kong, Hong Kong 999077, China; Department of Electrical and Electronic Engineering, The University of Hong Kong, Hong Kong 999077, China; Department of Mechanical Engineering, The University of Hong Kong, Hong Kong 999077, China; Department of Mechanical Engineering, The University of Hong Kong, Hong Kong 999077, China; Advanced Biomedical Instrumentation Centre Limited, Hong Kong 999077, China; Department of Electrical and Electronic Engineering, The University of Hong Kong, Hong Kong 999077, China; Department of Biomedical Engineering, The Chinese University of Hong Kong, Hong Kong 999077, China; Department of Biomedical Engineering, City University of Hong Kong, Hong Kong 999077, China; Center of Super-Diamond and Advanced Films (COSDAF), City University of Hong Kong, Hong Kong 999077, China; Hong Kong Centre for Cerebro-Cardiovascular Health Engineering, Hong Kong 999077, China; Department of Mechanical Engineering, The University of Hong Kong, Hong Kong 999077, China; Advanced Biomedical Instrumentation Centre Limited, Hong Kong 999077, China; Materials Innovation Institute for Life Sciences and Energy (MILES), The University of Hong Kong Shenzhen Institute of Research and Innovation (HKU-SIRI), Shenzhen 518057, China

**Keywords:** ultrathin hydrogel electronics, biomimetic materials, nanofiber frameworks, 3D conformability

## Abstract

Hydrogels are promising materials for constructing next-generation bioelectronics because of their excellent biocompatibility and mechanical compliance. Yet, creating robust and multifunctional hydrogel devices that conform to the surface of 3D organs remains challenging. Here, we report a biomimetic strategy for engineering ultrathin and ultrastrong hydrogel membranes as an advanced platform for organ-conformal bioelectronics. In these hydrogels, self-organized nanofiber networks confer strain-stiffening characteristics with a phenomenal combination of high mechanical strength (∼13.65 MPa), fracture toughness (∼21 573 J/m^2^), and low initial stiffness (∼600 kPa), which accommodates the construction of ultrathin membranes (∼10 μm thickness) reconciling mechanical robustness and 3D conformability. Theoretical simulations reveal unique strengthening mechanisms originating from the topological reconfiguration of fibrillar joints, indicating a widely applicable principle for designing soft composites involving 3D fibrillar networks. We show that various electronic components, including conducting polymers and wafer-fabricated microelectronic sensors, can be integrated on the ultrathin hydrogel membranes, providing means for multimodal physiological sensing and stimulation. These hydrogel membranes open paths to robust, functional and biocompatible interfaces with 3D soft organs and tissues, which are useful for epidermal electronics, implantable brain-machine interfaces, peripheral nerve stimulation, and many other bioelectronic applications.

## Teaser

Topologically reconfigurable fibrous networks enable hydrogel membranes that integrate exceptional mechanical robustness and ultrathin architectures, leading to stable and conformal bioelectronic interfaces with complex biological surfaces.

## INTRODUCTION

Developing advanced implantable bioelectronics requires soft functional materials that can naturally integrate with 3D organs and establish electrically addressable interfaces [1]. The enabled soft systems would provide critical capabilities for neural prosthesis [2], brain-machine communication [3,4], cardiac pacemakers and defibrillators [5], and many other applications [6,7]. Among many materials candidates, hydrogels present several advantages for the construction of implantable bioelectronics [8,9]. First, hydrogels involve polymeric networks retaining a significant amount of water, which resemble the ubiquitous structural motifs in living organisms [10]. Their porous structures allow mass transport, presenting minimal impacts on nutrient diffusion or biochemical signaling [11]. Second, the stiffness of hydrogels can be tuned within a range from kPa to MPa, similar to those of biological tissues [12]. Their mechanical compliance minimizes physical irritation or the risk of interfacial delamination [13]. Third, hydrogels could be chemically functionalized for controlled bio-interactions, providing a means for

drug delivery, anti-infection, mitigation of biofouling, interfacial adhesion, or other biomedical functions [14].

For practical applications, hydrogel-based bioelectronics should exhibit high flexibility in order to integrate with delicate 3D tissues such as the cortex and peripheral nerves [15]. On the other hand, mechanical robustness at the device level is essential for manufacturing, operation, and long-term reliability. However, these key attributes are often contradictory [16]. For instance, hydrogel devices with a thickness of ∼0.4–10 μm and a modulus of ∼50–1500 kPa achieved a seamless contact with human skin, serving as a conformal interface for electrophysiological measurements [17–19]. However, these ultrathin membranes have a low fracture strength of several hundred kPa, making it difficult to manipulate or achieve long-term stability under repeated body motion [20]. On the other hand, strengthening and toughening approaches for hydrogels, such as incorporating multiple networks [21,22], orientation alignment [23,24], and microcrystallization [25,26], may not be suitable for the fabrication of bioelectronic devices due to their specific processing requirements.

Another challenge with the hydrogel bioelectric interface lies in biological and electronic functionalization, as well as being processed into complex systems. Recently reported intrinsically electroconductive hydrogels [27,28] are not compatible with established microfabrication techniques because their processing usually involves chemical and thermal treatment [8]. Therefore, the resultant devices rarely present a high-density sensor array suitable for multimodal measurements of electrophysiology, biochemistry, temperature, mechanics, or in other domains.

Taken together, these limitations underscore the need for strategies that can simultaneously ensure mechanical robustness, tissue-like compliance, and compatibility with electronic functionalization. Therefore, rational material design is essential to achieve ultrathin hydrogel-based devices that integrate robustness with tissue-like compliance, enabling reliable 3D conformal bioelectronic interfaces. In natural biological tissues, evolutionary processes created soft membranes conformally integrated with diverse 3D organs. As exemplified by pericardium [29], epineurium [30], meninges [31], and dermis [32,33], these water-rich biological membranes exhibit exceptional mechanical strength for structural protection while retaining excellent thinness and deformability. A common feature in these membranes is the interconnected collagen fibrils which present excellent load-bearing characteristics and non-linear mechanical responses [34–36]. Although extensive engineering efforts have been devoted to the fabrication of microfibrillar materials mimicking natural soft tissues [26,37,38], achieving desired microstructural interactions and macroscopic properties matching those of natural tissues remains difficult. We recently developed a class of composite hydrogels featuring self-assembled microfibrillar networks, which exhibit structures and properties similar to those of natural load-bearing soft tissues [23,39]. Our previous work revealed that microstructural features of fibrillar networks, including nodal connectivity and nodal strength, synergistically governs the mechanical properties [40]. These insights inspired us to pursue designs toward natural-membrane-mimetic ultrathin hydrogel bioelectronic interfaces.

**Figure 1.**
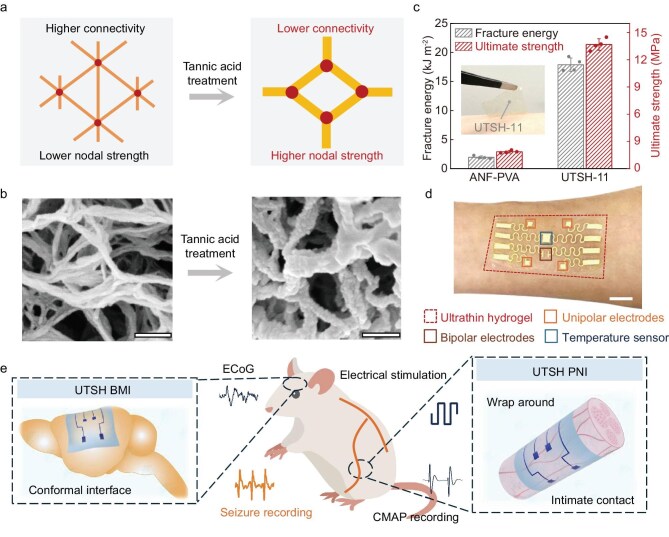
Design and structural characteristics of UTSHs. (a) Schematics illustrating the structural changes from aramid nanofibers-polyvinyl alcohol (ANF-PVA, left) to aramid nanofibers-polyvinyl alcohol-tannic acid (ANF-PVA-TA, right), showing stronger fibrillar joints and reduced nodal connectivity after the incorporation of tannic acid (TA). (b) SEM images of ANF-PVA (left) and ANF-PVA-TA (right) networks. Scale bar: 200 nm. (c) Comparison of fracture energy and ultimate tensile strength between the ANF-PVA hydrogel and the ultrathin and strong hydrogel (UTSH-11, containing 11 wt.% TA) with the same solid fraction. The inset shows a photograph of UTSH-11 attached to human skin. (d) Photograph of wafer-fabricated stretchable electronics integrated on UTSH-11 as an epidermal sensing platform. Scale bar: 1 cm. (e) Schematics of UTSH-based electronics as implantable systems, including brain-machine interfaces (BMI) and peripheral nerve interfaces (PNI).

Here, we introduce a microstructural tuning strategy to further optimize the biomimetic microfibrillar networks, enabling the engineering of hydrogel membrane devices as robust and conformal bioelectronic interfaces. Specifically, we employed polyphenol crosslinkers to enhance fibrillar binding and reduce nodal connectivity (Fig. 1a and b). This led to orders-of-magnitude improvements in mechanical properties at a constant solid content (Fig. 1c), without compromising softness. The resultant microfibrillar hydrogels exhibit outstanding mechanical strength and toughness combined with low initial modulus and strain-stiffening behaviors. These properties are critically important for the realization of ultrathin and strong hydrogels (UTSHs) mimicking biological membrane tissues that conformally envelop soft 3D organs. The processability of UTSHs further allows integration of conducting polymers and wafer-fabricated stretchable sensors into multifunctional bioelectronic membranes (Fig. 1d). These devices create robust, functional, and biocompatible interfaces with 3D soft organs and tissues (Fig. 1e), which are useful for epidermal electronics, implantable brain-machine interfaces (BMI), peripheral nerve interfaces (PNI), and many other bioelectronic systems.

## RESULTS AND DISCUSSION

### Designs and mechanical properties of UTSHs

**Figure 2.**
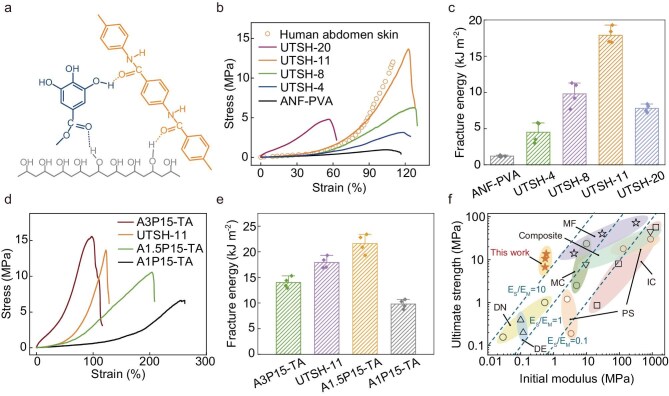
Mechanical properties of UTSHs. (a) A schematic of intermolecular interactions among ANF, PVA, and TA involved in UTSHs. (b) Comparison of strain-stress curves for UTSHs with various TA contents (ranging from 0 wt.% to 20 wt.%) and human skin. (c) Fracture energies of UTSHs with different TA contents. (d and e) Stress–strain curves (d) and fracture energies (e) of ANF-PVA-TA with various initial ratios between ANF and PVA (from 1:15 to 3:15). (f) Ultimate tensile strength and initial modulus of A1P15-TA, A1.5P15-TA, and A2P15-TA (UTSH-11), compared with those of hydrogels featuring composite composition, dense entanglements (DE), ionic crosslinking (IC), double networks (DN), microcrystals (MC), microfibers (MF), and phase separation (PS), all known for their superior mechanical performance (see details in Table S3). The UTSHs exhibited a high *E*_S_/*E*_M_ ratio, indicating their outstanding combination of mechanical strength and softness.

A key component in UTSHs is a self-organized microfibrillar network involving aramid nanofibers (ANFs) interlaced with soft polymers (e.g. polyvinyl alcohol, or PVA). The solvent-based processing generates a highly interconnected 3D architecture with fibrillar joints strongly welded through hydrogen-bonded PVA chains (Fig. S1) [39]. Although these microfibrillar hydrogels exhibit remarkable mechanical characteristics, further engineering of their properties satisfying their application in ultrathin membrane devices remains difficult, since enhancing mechanical strength for a fibrillar network is usually associated with an increase of stiffness or solid fraction. To address this issue, we introduce additional composition into the hydrogels to facilitate topological reconfiguration of the fibrillar network, which enables convenient tuning of their stiffness, toughness, and non-linear mechanics even at a constant solid fraction. TA is a polyphenol presenting abundant functional groups for non-covalent intermolecular interactions. It provides reconfigurable crosslinking with both PVA and ANFs, without compromising the integrity of the bridging interactions between them (Fig. 2a). It is conceivable that incorporation of TA into ANF-PVA hydrogels may lead to an important microstructural reconfiguration of the fibrillar network accompanied by pronounced changes of macroscopic mechanics. First, the enhanced attraction between PVA chains facilitates the bundling of fibrils, which reduces nodal connectivity (or coordination number) and increases the diameter of fibrils and mesh size (Fig. S2). Second, the binding strength of the fibrillar joints will further increase with the extensive crosslinking between soft PVA chains and jointing of fibrils. Third, the contraction of crosslinked PVA may result in bending or crimping of ANFs, which reduces the initial tensile modulus and imparts strain-stiffening behaviors to the network. Indeed, many of these microstructural features are evidenced by examination through scanning electron microscopy (SEM) (Fig. 1b and Fig. S3). The enhanced hydrogen bonding by TA is confirmed by Fourier transform infrared spectroscopy (FTIR), which shows significant red shifts in the C=O stretching band of ANF and the O–H stretching band of PVA after the incorporation of TA (Fig. S4). Furthermore, we observed a geometric shrinking of ANF-PVA hydrogel membranes when immersed in aqueous solutions of TA (Fig. S5), indicating the strengthened intermolecular interactions in the presence of TA.

Incorporation of TA in the UTSHs leads to a dramatic enhancement of mechanical properties as compared with hydrogel membranes based on ANF-PVA only. We tested UTSHs containing various amounts of TA while maintaining a constant initial ANF to PVA weight ratio of 2:15. The UTSHs were prepared by doctor-blading the liquid precursor of ANF-PVA onto a planar substrate followed by a solvent exchange to generate solid hydrogel membranes. The TA content was customized by immersing the ANF-PVA hydrogel membranes into aqueous solutions of TA with various concentrations (Fig. S6). The thinness of the membranes (∼10 μm) minimizes the non-uniform distribution of TA [41]. We found that the ultimate tensile strength of UTSH increases dramatically with increasing TA content, accompanied by a pronounced strain-stiffening behavior. We note that components in UTSH, such as ANF and TA, are indispensable for the strain-stiffening properties (Fig. S7 and Fig. S8). UTSH involving 11 wt.% of TA (denoted as UTSH-11) exhibits a remarkable tensile strength of 13.65 MPa while retaining a low initial modulus of 600 kPa and a water content of 74 wt.%, similar to those of natural skin (Fig. S9). It presents skin-mimetic non-linear mechanical responses as well (Fig. 2b). These properties are crucial for the construction of membrane devices reconciling mechanical robustness and high deformability. As compared with ANF-PVA hydrogels, UTSH-11 exhibits an enhancement of mechanical strength by 780% even with an identical solid fraction (Fig. S10 and Table S1). The toughness of UTSH, as characterized by the fracture energy during tearing, also reaches a remarkable value of 17 908 J/m^2^ in UTSH-11, which is 1490% higher than that of ANF-PVA hydrogels (Fig. 2c). The stretchability of UTSHs with moderate TA content falls in a range from 118% to 123%, indicating their similar network topology. Further increase of TA content to 20 wt.% leads to a decrease in strength and stretchability of the hydrogel, possibly due to the disruption of 3D fibrillar architecture with TA dominating the solid content. We note that aerogels derived from UTSHs and ANF-PVA also exhibit differences in strength and non-linear behaviors, indicating common deformation mechanisms originating from the fibrillar networks (Fig. S11). The hysteresis observed during cyclic loading indicates the viscoelasticity and energy dissipation capability of UTSHs, with a low residual plastic deformation ∼2.1% even after 100 cycles at 10% maximum strain (Fig. S12 and Table S2). Notably, the unique strain-stiffening behavior was retained after cyclic tensile loading even at a maximum strain of 50% (Fig. S13), which may be attributed to the structural stability of the microfibrillar network (Fig. S14). The strength and modulus did not exhibit any decrease following cyclic loading, underscoring the robustness of the UTSHs.

In addition, the mechanical properties of UTSHs can be tuned by adjusting the ratio between ANF and PVA in the hydrogel precursor, in which a slight decrease of ANF content would lead to an increase of stretchability and a decrease of tensile strength (Fig. 2d). For example, as compared with UTSH-11, reducing ANF content by 25% (referred to as A1.5P15-TA in Fig. 2d) can enhance the stretchability to 203% along with an increase of fracture energy (up to 21 573 J/m^2^) and a slight decrease of tensile strength (to 10.6 MPa) (Fig. 2e and Fig. S15). Notably, in comparison with other hydrogel systems renowned for their mechanical performance, including those based on dense entanglement (DE), ionic crosslinking (IC), double-network (DN) structures, and other reinforcement strategies, UTSH demonstrates a significantly higher fracture strength to modulus (*E*_S_/*E*_M_) ratio. These mechanical characteristics of UTSH, in combination with its excellent biocompatibility and processibility for microelectronic fabrication, are critically important for the engineering of conformal bioelectronic interfaces (Fig. 2f and Table S3).

### Theoretical simulations and mechanistic analyses

**Figure 3.**
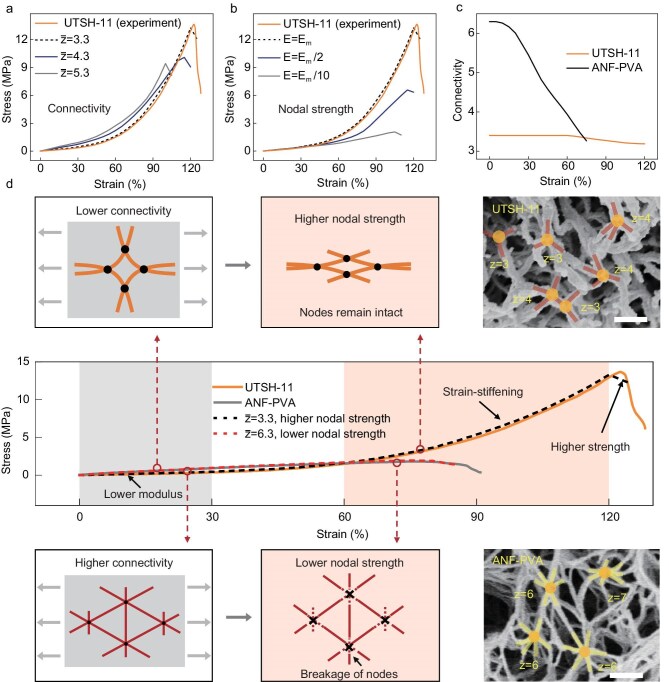
Theoretical simulation and mechanistic analysis. (a) Effects of mean nodal connectivity (*z̅*) on the tensile response of simulated 3D networks. The simulated results for *z̅* = 3.3 match well with the experimental observations (dashed line) of UTSH-11. (b) Effect of mean nodal strength, represented by the binding energy, on the simulated tensile responses of 3D networks with a mean nodal connectivity of *z̅* = 3.3. (c) Comparison of the average connectivity of a simulated ANF-PVA network and an ANF-PVA-TA network (UTSH-11) as a function of imposed strain. (d) Comparison of experimental (solid line) and simulated (dashed line) tensile stress-strain curves of ANF-PVA and UTSH-11. The simulated mechanical responses of *z̅* = 3.3 with a higher nodal strength, and *z̅* = 6.3 with lower nodal strength, align well with ANF-PVA and UTSH-11, respectively (see details in Table S4). The schematic illustrates the state of the ANF-PVA network (lower) and UTSH-11 (upper) during the initial (gray region) and final (flesh-colored region) tensile stages. The SEM images show the initial nodal connectivity for ANF-PVA and UTSH-11. Scale bar: 200 nm.

We developed a computational model to study the mechanical responses of 3D fibrillar networks in relation to their microstructural features, providing insights for the general design of soft fibrillar composites (Fig. S16 and Table S4). In particular, random networks with various levels of nodal connectivity, mesh size, and fibril diameter can be constructed with linear and rotational springs crosslinking each pair of intersecting fibrils. Individual fibrils are modeled as cylindrical segments with a finite modulus, which can withstand stretching, bending, and twisting. The rigidity and strength of the fibrillar joints are customized by adjusting the spring constants of the crosslinkers and their critical strain energy before failure (Fig. S17). Our previous studies on microfibrillar aerogels demonstrate that increasing the connectivity and binding strength of fibrillar joints leads to a dramatic enhancement of macroscopic stiffness and strength, even at a constant solid fraction [40]. However, the distinct mechanical responses of UTSHs require careful adjustments of the computational model. Indeed, the observed strain-stiffening of UTSHs under tension is quite different from the strain-softening behaviors of aerogels, which could be related to their distinct topological features and micromechanical characteristics. Although the exact intermolecular interactions in UTSHs are difficult to simulate, we attempt to recapitulate the realistic stress-strain responses of UTSHs by tuning the parameters in the 3D fibrillar model (Fig. 3a and Fig. S18). First, the experimentally observed decrease of nodal connectivity is configured in the computational model, in which we use an average connectivity of $\bar{z}$ = 3.3 to simulate the fibrillar network in UTSH-11, as compared to $\bar{z}$ = 6.3 for the ANF-PVA hydrogel without TA. Second, the strengthened nodal interactions induced by TA would require an increase in the binding energy of crosslinkers (60 nN·μm as compared to 10 nN·μm for ANF-PVA). The high nodal strength also reflects the influence from plastic reconfiguration of the hydrated polymers. Third, the length and diameter of fibrils are tuned to approximate experimental observations while retaining a constant solid fraction of 26%, similar to that of UTSH-11. With these adjustments of the model, our simulation successfully replicated the tensile stress-strain responses of UTSH-11 observed experimentally (Fig. 3a).

The computational model allows for thorough investigation of various microstructural features and their impacts on macroscopic mechanics while ruling out the variation of solid content. We studied the effects of network topology by tuning the nodal connectivity from 3.3 to 5.3, while having other mechanical parameters similar to those of UTSH-11. The simulated tensile responses show that nodal connectivity has a major influence on the initial modulus and strain-stiffening behaviors (Fig. 3a). At low strains, lower connectivity affords reorientation of the fibrils without breakage of their connections, leading to a lower modulus. This phenomenon is similar to that dictated by the Maxwell theory for a pin-connected truss network, in which the overall structural rigidity occurs only when the nodal connectivity is above a threshold value [42]. We note that our computational model captures the realistic stiffness of the network with nodal connectivity even below the rigidity percolation threshold in Maxwell theory. As the strain increases, the aligned fibrils and their crosslinks start to bear mechanical loads by stretching (Fig. S19), leading to a significant increase of stiffness. In this regard, the binding energy at the fibrillar joints should have major effects on the overall mechanical response. Our simulation shows that reducing the nodal binding energy leads to a dramatic reduction in tensile strength along with diminishing strain-stiffening characteristics (Fig. 3b), corresponding to the early onset of nodal breakage compromising the stretching of fibrils. On the other hand, nodal strength has little effect on the initial tensile modulus, suggesting that early-stage deformation is primarily governed by reorientation of fibrils in the low-connectivity network rather than nodal fracture.

Our computational model also replicates the mechanical behaviors of ANF-PVA hydrogels for comparison with UTSH-11 (Fig. 3c and d). Strain-stiffening behavior is absent in ANF-PVA since the high-connectivity network does not allow extensive re-orientation of fibrils without breaking their connections. Our simulation shows a gradual decrease of average nodal connectivity in ANF-PVA under tension (Fig. 3c), which contrasts with UTSH-11 exhibiting stable nodal connectivity even at 60% imposed strain. Based on the simulation results, we argue that the outstanding strength and non-linear mechanical behaviors of UTSHs can be attributed to the synergy between high binding energy and low connectivity of the fibrillar joints, which originate from the microstructural reconfiguration induced by TA. Indeed, tuning of topological features and nodal mechanics of a fibrillar network could enable a powerful approach to the engineering of macroscopic properties for diverse materials.

### Applications in conformal bioelectronics

**Figure 4.**
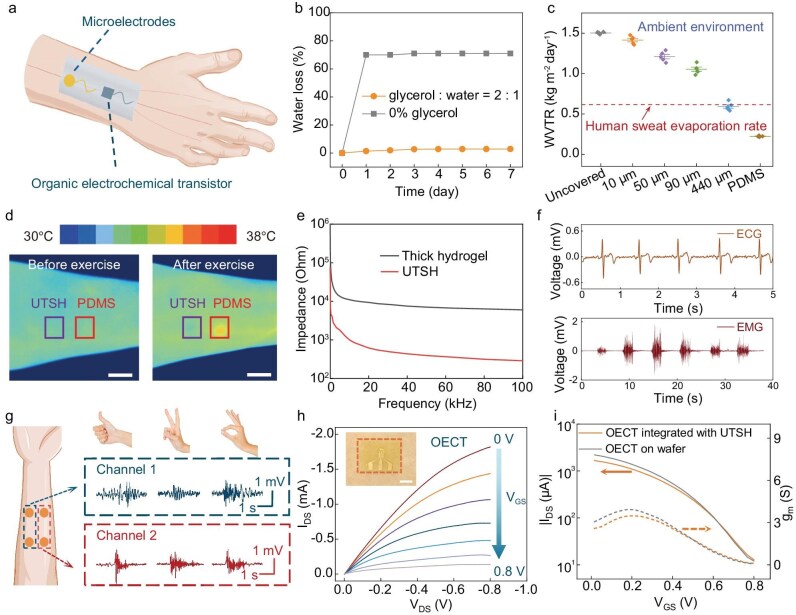
Epidermal electronics based on UTSHs. (a) A schematic illustrating a conformal UTSH electronic membrane attached to the skin. (b) Water retention performance of UTSHs modified with glycerin, tested for 7 days under ambient environment. (c) Comparison of water vapor transmission rates (WVTR) for an uncovered bottle, a bottle covered with a 100-μm-thick PDMS film, and bottles covered with ANF-PVA-TA hydrogels with various thicknesses (from 10 μm to 440 μm). (d) Infrared thermal images before (left) and after (right) 20-min running exercise of a forearm covered with glycerin-modified UTSHs and PDMS, demonstrating the superior breathability of UTSHs compared to PDMS film. Scale bar: 2 cm. (e) Skin-electrode interfacial impedance measured with devices based on ultrathin and thicker hydrogels. (f) ECG (top) and EMG (bottom) data collected using wafer-fabricated gold micro electrodes integrated on UTSHs. (g) Gesture recognition achieved via EMG signals. (h) Output curves of OECTs transferred to UTSHs (the channel width (*W*) is 400 μm, and the channel length (*L*) is 40 μm, *W*/*L* = 10), where *V_ds_* values corresponded to 0 V, 0.2 V, 0.3 V, 0.4 V, 0.5 V, 0.6 V, 0.8 V. Scale bar: 1 cm. (i) Transfer curves of the OECTs integrated on UTSHs with *V_GS_* value of −0.6 V. The transconductance (*G_m_*) is calculated from the derivative of the transfer curves.

The excellent mechanical properties, manufacturability, and scalability of UTSHs allows incorporation of multifunctional soft electronics and chemical modifications for the construction of bioelectronic interfaces (Fig. 4a and Fig. S20). For instance, hybrid arrays of microsensors and stimulators can be fabricated on a planar substrate and picked up by a water-soluble tape [43]. After plasma-activation of the polymeric encapsulation layer, the liquid precursor of UTSHs can be cast on the tape-supported bioelectronic devices followed by solidification in water and release of the tape (Fig. S21). This process generates robust interfacial integration between bioelectronic components and the hydrogel membrane, with an adhesion energy of ∼12 J/m^2^ (Fig. S22). In another scheme, conducting polymers can be patterned into UTSHs to form low impedance bioelectrodes. Specifically, polypyrrole (PPy) can be polymerized around the microfibrillar framework in an aqueous environment [41], enabling efficient electron-transport pathways with an ultralow percolation threshold (∼1 wt.%) and an electrical conductivity on the order of 29 S cm^−1^. Patterns of electrodes and interconnects can be defined by a mask allowing for area-selective polymerization. Furthermore, the adhesion between UTSHs and biological tissues can be promoted by a thin coating (∼3 μm thickness) of polyacrylic acid (PAA) on the surface of UTSHs. To avoid water loss during long-term application in the air, glycerin can be infiltrated into UTSHs, which ensures a minimal change of mass (∼2.9%) even after a 7-day exposure in the atmosphere (Fig. 4b). The abundant water in UTSHs is also advantageous for maintaining a high level of skin hydration (Fig. S23). Indeed, incorporation of these and other functional components does not compromise the favorable mechanical characteristics of UTSHs (Fig. S24), allowing for customization of conformal bioelectronics systems tailored for diverse applications.

Epidermal electronics based on UTSHs exhibit outstanding performance as compared to devices based on other substrates (Fig. 4a). The adhesion energy between a PAA-coated UTSH device and porcine skin reaches 6.11 J/m^2^, which is 32 times stronger than that from a PDMS substrate (Fig. S25). The adhesion remained stable over 48 hours, indicating its potential for continuous usage. The intrinsic permeability of the porous network [44], coupled with ultrathin membrane structures, ensures excellent breathability of UTSH-based epidermal electronics. A high water vapor transmission rate (WVTR) of 1.42 kg m^−2^ day^−1^ is achieved with a 10-μm UTSH membrane (Fig. 4c), which exceeds the human sweat evaporation rate (∼0.6 kg m^−2^ day^−1^) [18]. The excellent breathability was further validated through on-skin experiments. We measured skin temperature on a forearm covered with UTSH and PDMS, before and after 20-min running exercise. In contrast with the PDMS film, the UTSH caused a lower temperature increase post-exercise (Fig. 4d).

Regarding electronic components, the conformability of UTSH-based devices ensures a low interfacial electrical impedance between electrodes and human skin (Fig. 4e). Stable ECG and EMG signals with precise details were recorded by placing the UTSH-based metal electrodes on the forearm (Fig. 4f). Multi-channel measurements capture unique patterns associated with the contraction of different muscle groups, which can be exploited for gesture recognition (Fig. 4g). The resistive sensor assembled onto the UTSHs exhibits a stable linear response to temperature changes, suggesting its utility as wearable temperature sensors (Fig. S26). We also demonstrate the integration of organic electrochemical transistors (OECTs) on UTSHs (Fig. 4h and Fig. S27). Compared to the as-fabricated OECTs on wafers, devices transferred onto UTSHs exhibit minimal changes in the transfer curves and transconductance (Fig. 4i). With the gate electrodes modified with glucose oxidase (GOx), the UTSH-based OECTs can respond to the variation of glucose content across a wide concentration range (10⁻^6^ to 10⁻^1^ M) (Fig. S28), indicating potential applications as wearable biochemical sensors.

**Figure 5.**
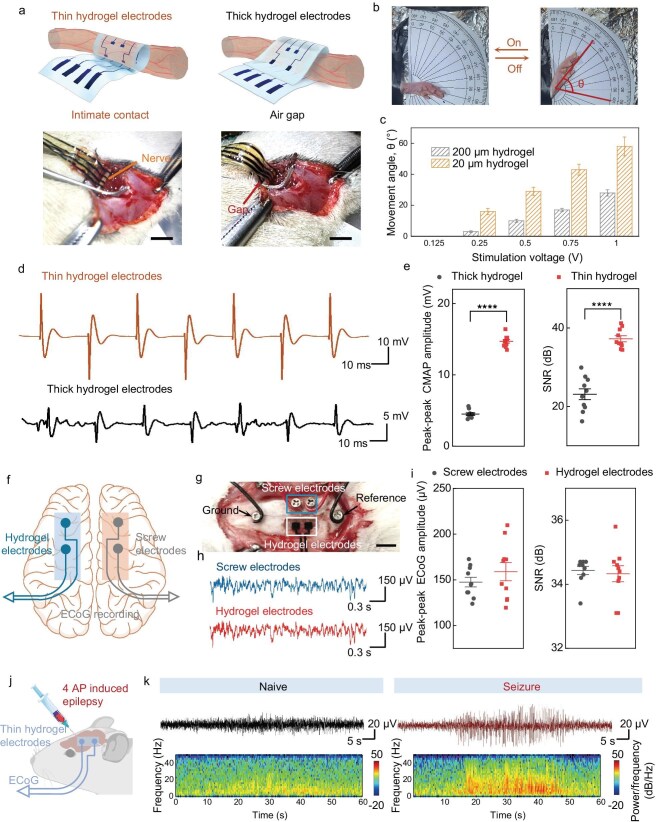
Conformal neural interfaces based on UTSHs. (a) Schematics and corresponding photographs of UTSH-polypyrrole (PPy) and thick hydrogel-PPy electrodes interfaced with rat sciatic nerves. Seamless contact is achieved by wrapping a UTSH-based device around the sciatic nerve. Scale bar: 5 mm. (b) Photographs showing the motion of the hindlimb when sciatic nerve stimulation is off (left) and on (right). (c) Comparison of hindlimb movement angles induced by the stimulation with UTSH-PPy and thick hydrogel-PPy electrodes. (d) Representative compound muscle action potentials (CMAPs) recorded with electrodes placed at the position where the gastrocnemius muscle had its maximum diameter, under the sciatic nerve stimulation using UTSH-PPy and thick hydrogel-PPy, respectively. (e) Comparison of the amplitude (left) and signal-to-noise ratio (SNR, right) values of CMAPs induced by the stimulation with UTSH-PPy and thick hydrogel-PPy electrodes. (f) A schematic of ECoG recording using non-destructive UTSH-PPy electrodes and intrusive commercial screw electrodes. (g) Optical images of non-destructive UTSH-PPy electrodes and intrusive screw electrodes placed on the cerebral cortex. Scale bar: 5 mm. (h) Representative ECoG signals recorded by UTSH-PPy and screw electrodes. (i) Comparison of the amplitude (left) and SNR (right) values of ECoG signals recorded by UTSH-PPy and screw electrodes. (j) A schematic of monitoring epileptic seizures with ECoG recorded with UTSH-PPy electrodes. Epilepsy is induced via injecting 4-AP. (k) Power spectra of ECoG signals recorded by UTSH-PPy electrodes before (left) and after (right) seizures.

Owing to the superior deformability and inherent biocompatibility of hydrogels compared to conventional polymers, the UTSH-based electronic membranes can form a seamless contact with nerve tissues without the need for adhesives or sutures, thereby potentially reducing nerve damage compared to previously reported polymer electrodes or adhesive-based hydrogel designs [45]. A device with a thickness of ∼20 μm enables seamless contact with rat sciatic nerves without the need for adhesives or sutures (Fig. 5a). In contrast, application of a device based on a thick hydrogel membrane (∼200 μm thickness) inevitably leads to a gap between the electronics and the nerve tissue, which could compromise effective stimulation and recording of signals. Using these hydrogel membrane electronics, we applied electrical stimulation (40 Hz square wave) to the sciatic nerve and recorded compound muscle action potentials (CMAPs) from the gastrocnemius and tibialis anterior muscles, as well as the motion of the hind limb. Compared to stimulation with thick hydrogel electrodes, the use of UTSH electrodes leads to significantly higher amplitude and signal-to-noise ratio for CMAPs, larger angle of hind limb motion, and lower voltage threshold for effective stimulation (Fig. 5b–e and Fig. S29). In another experiment, we applied UTSH-based hydrogel electrodes on the dura mater for intracranial recording of electrocorticography (ECoG). The conformal device captured high quality signals comparable to those recorded with commercial screw electrodes (Fig. 5f, i and Fig. S30). We also demonstrate its use for intraoperative monitoring of epilepsy, which is important for guiding surgical intervention (Fig. 5j and k).

**Figure 6.**
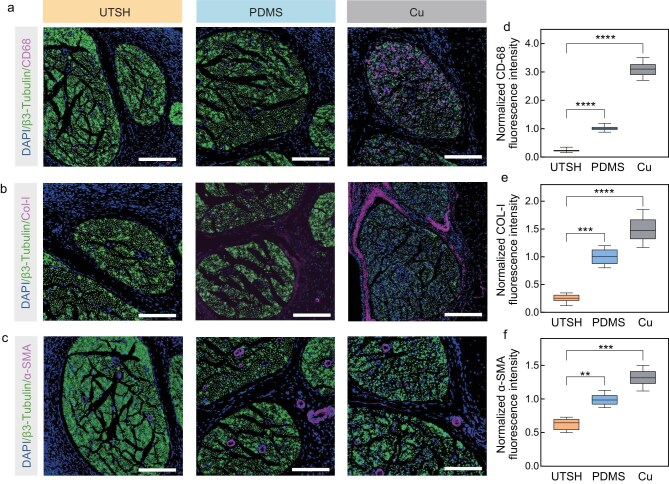
*In-vivo* biocompatibility of UTSHs. (a–c) Representative immunofluorescence images of rat sciatic nerves wrapped with UTSH, PDMS, and Cu films after 14 days of implantation. Cell nuclei are stained with DAPI (blue). Green fluorescence corresponds to the expression of a neuron marker β3-Tubulin. Purple fluorescence corresponds to macrophages (CD68, a), collagen (Collagen I, b) and fibroblasts (α-SMA, c), respectively. Scale bars: 200 μm. (d–f) Normalized fluorescence intensity plots of CD-68 (d), COL-1 (e), and α-SMA (f); *n* = 30, **P < 0.01, *** *P* < 0.001, ****P < 0.0001. The implant based on UTSH leads to better neuron health and lower foreign body responses as compared to PDMS and Cu.

We evaluated the biocompatibility of UTSH-based devices as long-term implants. In a preliminary cell experiment, NIH 3T3 fibroblasts cultured on top of UTSHs show rapid proliferation and high survivability, indicating little cytotoxicity of UTSHs (Fig. S31). We also evaluated the *in-vivo* biocompatibility of UTSHs by implanting them surrounding the sciatic nerves of rats for 14 days (Fig. S32). Film devices based on polydimethylsiloxane (PDMS) and copper (Cu) were implanted as controls. Immunofluorescence staining of various markers was performed to assess neuron health and foreign body responses (Fig. 6a–c). Compared to the controls, implanted UTSHs lead to a higher expression of β3-Tubulin (an indicator of neuron health) (Fig. S33) and lower expression of CD68 (a marker for macrophages) (Fig. 6a and d), α-smooth muscle actin (or α-SMA, a marker for fibroblasts) (Fig. 6b and e), and collagen-I (Fig. 6c and f) at the nerve-implant interface. Furthermore, UTSH remains stable after 14 days of immersion in PBS, highlighting its potential as a long-term implant (Fig. S34). These results demonstrate that implants based on UTSH are conducive for the preservation of neuron health and minimization of adverse immune responses.

## CONCLUSION

In summary, we have demonstrated a biomimetic strategy for the construction of hydrogel membrane devices as robust and organ-conformal bioelectronic systems. In these hydrogels, the engineering of topological features of fibrillar networks creates a new dimension for the tuning of mechanical properties of materials, which leads to favorable characteristics not achieved with other hydrogel systems. The mechanistic insights could be generalized for the design of various soft composites involving fibrillar networks. Exploiting other assembly approaches for the customization of nodal connectivity or other microstructural features would further expand the achievable properties of materials, which could be useful for the engineering of tissue scaffolds, energy devices, filtration membranes, lightweight structures, and for many other applications. From the manufacturing standpoint, the robust microstructures and simple processing steps of UTSHs affords reliable fabrication of conformal membrane devices and integration with diverse functional components. Further incorporation of gene therapy vectors, drug delivery components, microfluidics, or cell-electronic hybrids would enhance the bioactivity of the implantable system, providing a versatile platform for advanced diagnostics and therapies.

## METHODS

### Fabrication of UTSHs

ANF-PVA hydrogels were prepared using our previously reported approach [39]. Briefly, 2 g of Kevlar para-aramid pulp and 2 g of KOH were first dispersed in 100 mL of dimethyl sulfoxide (DMSO) under magnetic stirring for 7 days at 95°C to obtain a 2 wt.% ANF dispersion. Separately, 15 g of PVA (99%+, Sigma-Aldrich) was dissolved in 100 mL of DMSO under magnetic stirring for 3 days to create a 15 wt.% PVA solution. The 2 wt.% ANF dispersion was then mixed with the 15 wt.% PVA solution in DMSO at a 1:1 mass ratio. The resulting liquid precursor was blade-coated onto a stainless-steel substrate to a controlled thickness and then immersed in water for 24 hours to form the ANF-PVA hydrogel membrane. Afterward, the hydrogel was immersed in TA solution for 24 hours.

### Modeling the deformation of 3D networks

An FCC lattice with dimensions of 6$\sqrt 2 $  *l_c_* × 6$\sqrt 2 $  *l_c_* × 6$\sqrt 2 $  *l_c_* was used as the basis for the generation of random 3D fibrillar networks, where *l_c_* is the distance between two joints as proposed by Broedersz *et al.* [46]. In this model, the fibrils are simplified as cylindrical segments undergoing large stretching, bending, and twisting deformation. A periodic FCC network with connectivity of $\bar{z} = 12$ was generated. Filament segments between vertices were randomly removed to customize connectivity of the network.

Stochastic displacements were imposed on each node to increase the randomness of the network; the *l_c_* was adjusted to generate a fiber density consistent with the experimental observation. Additionally, we generated six random networks at each connectivity level in order to obtain statistics to ensure that the analysis and conclusions drawn from the simulations are sufficiently representative.

The deformation responses of the networks were captured using the finite element method. In this method, each fibril was modeled as a three-dimensional Reissner beam [47] capable of undergoing large rotation, stretching, bending, and twisting. Strain energy in the crosslinker was calculated:


\begin{eqnarray*}
{E}_c = \frac{1}{2}{k}_s\delta {l}^2 + \frac{1}{2}{k}_r\delta {\theta }^2,
\end{eqnarray*}

where *k_s_* and *k_r_* represent the stiffness of liner and rotational springs, *δl* is the separation of the joint nodes and *δθ* is the change in the relative angle between two fibrils. In addition, once the energy reaches a critical value, the joints will unbind. Finally, periodic boundary conditions were applied on each side of the simulation network. Detailed parameters adopted in our simulations are listed in Table S4. During the simulation, the uniaxial tensile test was applied and the stress was recorded.

### Mechanical characterizations

The tensile tests were conducted at room temperature by a mechanical tester (Zwick Roell) with a fixed strain rate of 100% per minute. The engineering stress–strain curves were obtained to determine the tensile moduli, which were calculated as the ratio of stress to strain at *ε* = 5%. For the determination of fracture energy, both notched and unnotched samples were subjected to tensile testing. The results obtained from the notched samples were compared with those from the unnotched samples to calculate the fracture energy using a well-established method [48].

### Statistical analyses

All experimental results were run in triplicate unless otherwise specified. Numerical data were expressed as means ± standard deviation (SD).

### Ethical statement

All animal experiments were conducted in accordance with the protocols approved by the Animal Ethical Committee of City University of Hong Kong (CityU) and Department of Health of Government of HKSAR ((22–78) in DH/HT&A/8/2/5 Pt.8). Male SD rats weighing between 200 and 300 g and aged 8 weeks were obtained from CityU and kept in the Laboratory Animal Research Unit (LARU) of CityU.

## Supplementary Material

nwag105_Supplemental_File
