# Supplementary material for: Ultrathin and ultrastrong hydrogel bioelectronic membranes
Source: Natl Sci Rev. 2026 Feb 12;13(8):nwag105. doi: 10.1093/nsr/nwag105 (PMC13116326; doi:10.1093/nsr/nwag105)
Supplement: nwag105_Supplemental_File [file nwag105_supplemental_file.pdf]

Supporting Information

Ultrathin and Ultrastrong Hydrogel Bioelectronic Membranes

Mingze Sun (孙铭泽)[1,3]†, He Zhang (张贺)[1,2]†, Xingdao He (何星道)[4]†, Xi Wei (魏茜)[5], Binbin Cui (崔彬彬)[6], Hao Huang (黄浩)[6], Hao Li (李昊)[1], Yuan Lin (林原)[1,2], Shiming Zhang (张世明)[6], Zhong Alan Li (李中)[7], Peng Shi (史鹏) [4,8,9]*, Lizhi Xu (徐立之)[1,2,10]*

[1] Department of Mechanical Engineering, The University of Hong Kong, Hong Kong SAR, China.

[2] Advanced Biomedical Instrumentation Centre Limited, Hong Kong SAR, China.

[3] Department of Cell Biology, Third Military Medical University, Chongqing, China.

[4] Department of Biomedical Engineering, City University of Hong Kong, Hong Kong SAR, China.

[5] School of Medicine, Wuhan University of Science and Technology, Wuhan, China.

[6] Department of Electrical and Electronic Engineering, The University of Hong Kong, Hong Kong SAR, China.

[7] Department of Biomedical Engineering, The Chinese University of Hong Kong, Hong Kong SAR, China

[8] Center of Super-Diamond and Advanced Films (COSDAF), City University of Hong Kong, Hong Kong SAR, China.

[9] Hong Kong Centre for Cerebro-Cardiovascular Health Engineering, Hong Kong Science Park, Hong Kong SAR, China.

[10] Materials Innovation Institute for Life Sciences and Energy (MILES), The University of Hong Kong Shenzhen Institute of Research and Innovation (HKU-SIRI), Shenzhen, China.

*Corresponding author. Email: xulizhi@hku.hk (L. X.) pengshi@cityu.edu.hk (P. S.)

† These authors contributed equally to this work

Content List:

1, Supplementary Figures and Captions

2, Supplementary Tables and Captions

3, References

Supplementary Figures and Captions

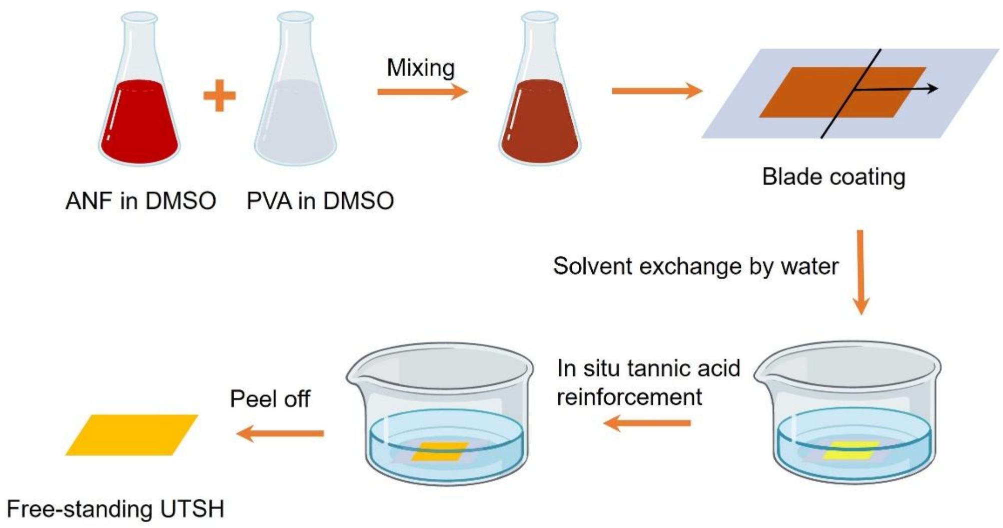

Fig. S1 A schematic of the fabrication processes for UTSHs. The fabrication of UTSHs is based on an ANF-PVA hydrogel membrane formed via a simple solution process. A mixed dispersion of ANF and dissolved PVA in DMSO is doctor-bladed onto a stainless-steel substrate, followed by soaking in water to form a self-organized 3D network. Next, TA was infused into the porous 3D network in an aqueous medium.

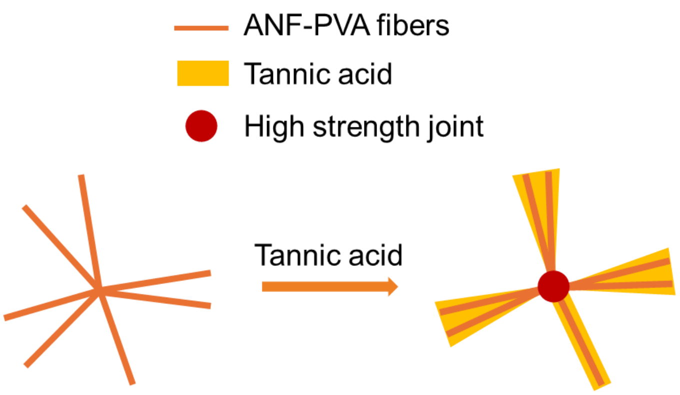

Fig. S2 A schematic of the topological regulation after adding TA. The introduction of TA can lead to bundling between nanofibers, thus increasing the diameter of fibrils and reducing the nodal connectivity.

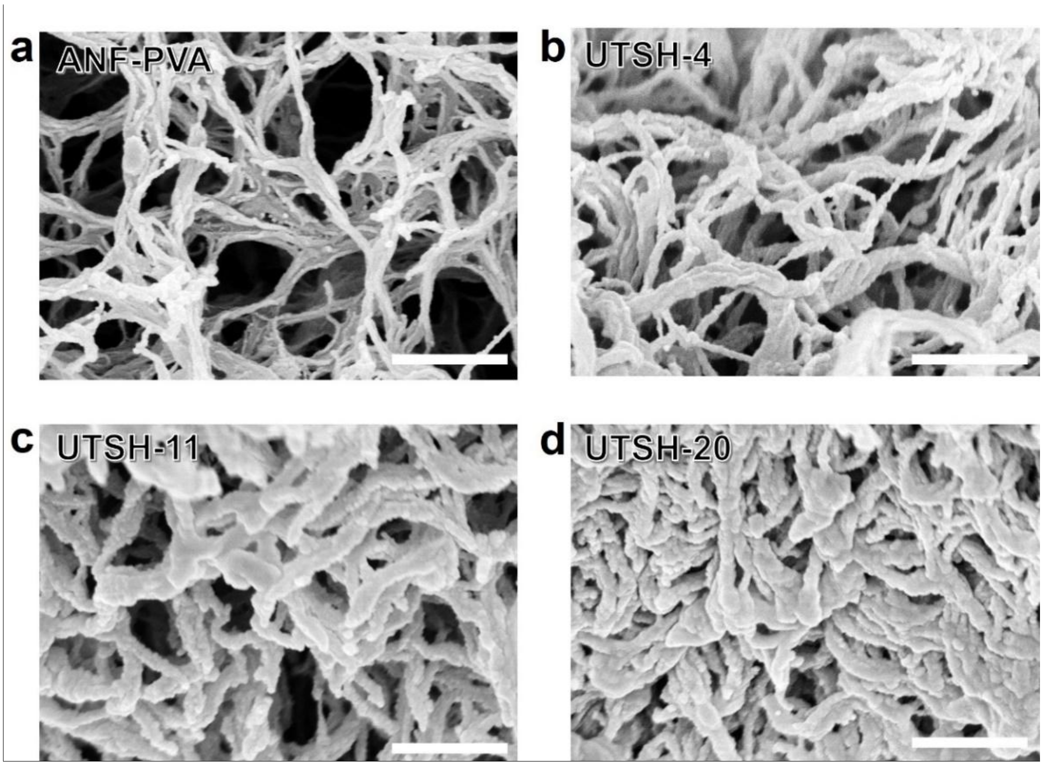

Fig. S3 SEM images of various hydrogels with different TA contents. (**a**) ANF-PVA. (**b**) UTSH-4. (**c**) UTSH-11. (**d**) UTSH-20. Scale bar: 400 nm. As TA content increased, fibers formed stronger joints and bundled together, resulting in increased fiber diameter and decreased nodal connectivity. However, when the TA content was too high, the porous network morphology was lost.

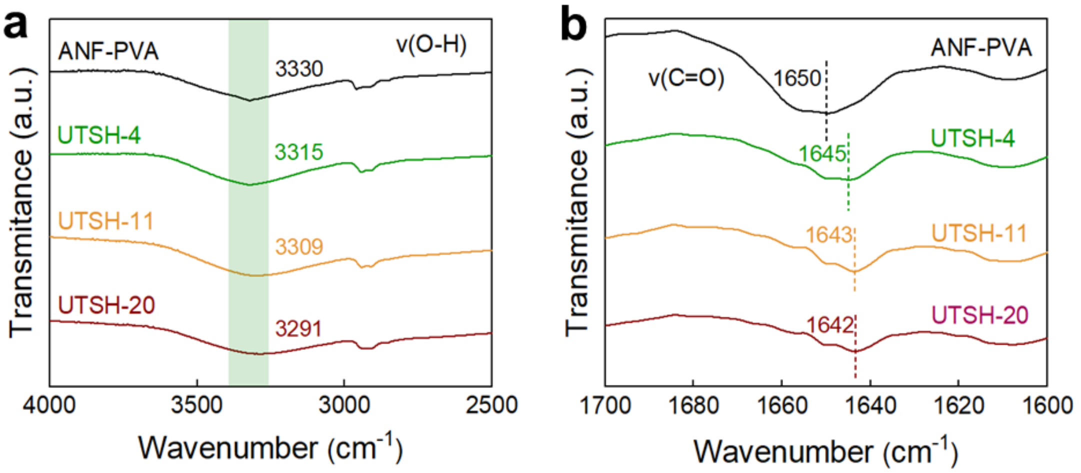

Fig. S4 Chemical structures characterized by FTIR. (**a-b**) Spectra of O-H (from PVA and TA) (**a**) and C=O (from ANF) (**b**) in ANF-PVA, UTSH-4, UTSH-11 and UTSH-20. With the increasing of TA contents, the C=O peak shifted from 1650 cm^{-1} to 1642 cm^{-1}, and the O-H peak shifted from 3330 cm^{-1} to 3291 cm^{-1}, reflecting the effects of hydrogen bonding.

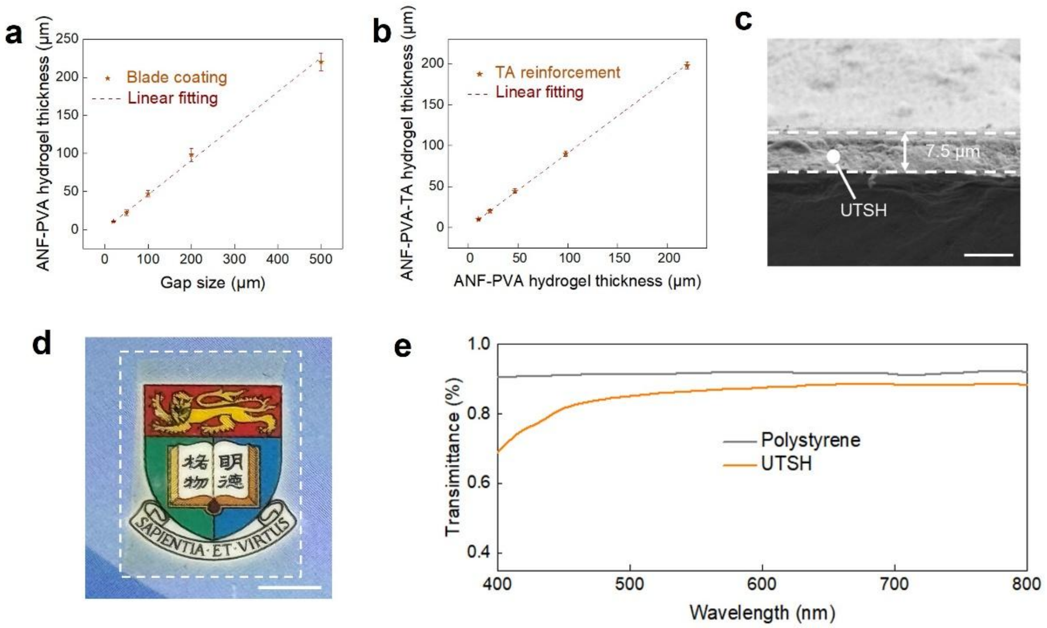

Fig. S5. Geometric shrinkage of hydrogel membranes. (**a**) The relationship between the thickness of ANF-PVA hydrogels and the gap size. The thickness of ANF-PVA precursors can be controlled by adjusting the gap size between the substrate and the blade. After immersion in water, the thickness of ANF-PVA precursors shrinks by approximately 50% due to the hydrogen bonding between ANF and PVA. (**b**) The relationship between the thickness of ANF-PVA-TA and ANF-PVA membranes. The hydrogel thickness further decreases by about 10% after immersion in a 2% TA solution. For instance, a hydrogel with an initial thickness of 10.5 µm shrinks to 9.8 µm after treatment, illustrating enhanced intermolecular interactions facilitated by TA. (**c**) Cross-sectional SEM image of a ~10-µm-thick final UTSH after shrinkage by TA reinforcement. Following CPD, the membrane thickness reduces to 7.5 µm, confirming its ultrathin property. Scale bar: 10 µm. (**d**) Optical image of a 10-µm-thick UTSH placed over the emblem of the University of Hong Kong. The emblem details are clearly visible, demonstrating the exceptional transparency achieved due to the ultrathin membrane thickness. Scale bar: 1 cm. (e) UV–vis transmittance spectra of UTSH and polystyrene culture plate.

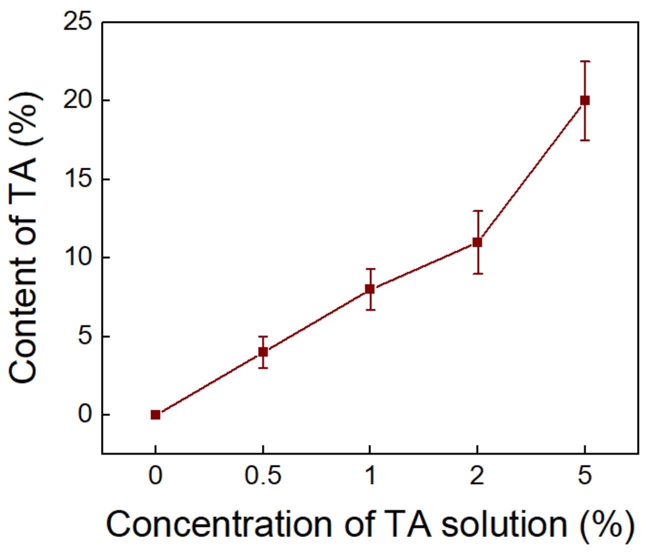

Fig. S6. TA content as a function of the concentration of TA solution used for immersion. The TA content in UTSHs can be adjusted by immersing the hydrogels in TA solutions of varying concentrations. As the TA solution concentration increases, the osmotic pressure rises, facilitating greater diffusion of TA into the network and leading to a gradual increase in TA content.

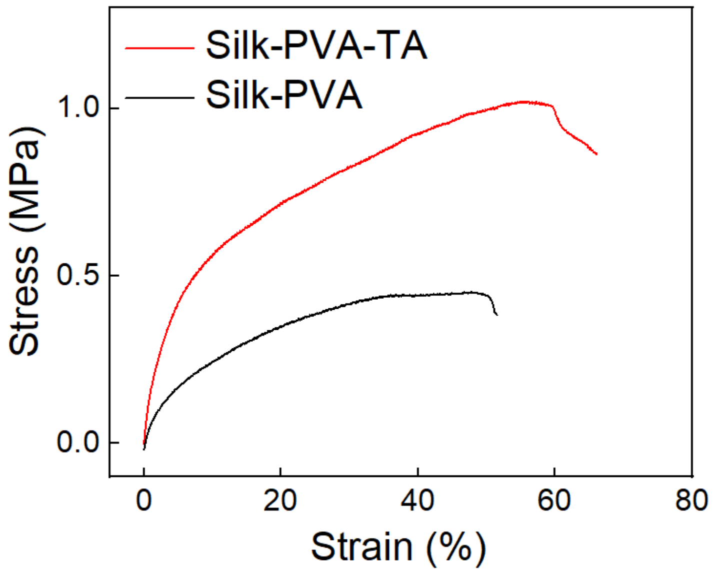

Fig. S7 Comparison of strain-stress curves between silk-PVA and silk-PVA-TA hydrogels. We explored silk fibers to composite with PVA. After incorporating TA, we observed an improvement in mechanical properties. However, although silk protein can form hydrogen bond interactions with PVA, the strength of the material remained limited, and no strain-stiffing behavior was detected. This may be due to the lower strength of silk fibers compared to ANFs, which constrained the performance of the final material.

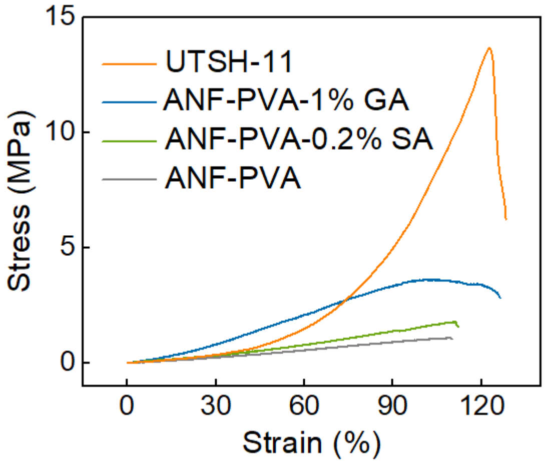

Fig. S8 Comparison of strain-stress curves between ANF-PVA, ANF-PVA-0.2 % (salicylic acid) SA, ANF-PVA-1 % gallic acid (GA) hydrogels and UTSH-11. We explored several small molecules with similar chemical structures, including gallic acid (GA) and salicylic acid (SA). Upon incorporating these molecules into the ANF-PVA hydrogel, we observed a similar enhancement in mechanical properties (Fig. S8), suggesting that they may exhibit a hydrogen bonding interactions within the hydrogel similar to that of TA. However, the enhancement effects of both GA and SA were less pronounced than that of TA, and no strain-stiffening behavior was observed, likely due to their limited solubility. GA has a maximum solubility of only 1% in water, while SA exhibits an even lower solubility of just 0.2%. These low solubility levels hinder the incorporation of sufficient amounts of GA and SA into the hydrogel matrix. As a result, the number of available hydrogen bonds is significantly reduced, leading to a diminished reinforcing effect. Therefore, a unique advantage of TA may be its high solubility in water, which allows it to fully diffuse into the ANF-PVA hydrogel, resulting in interaction and strengthening.

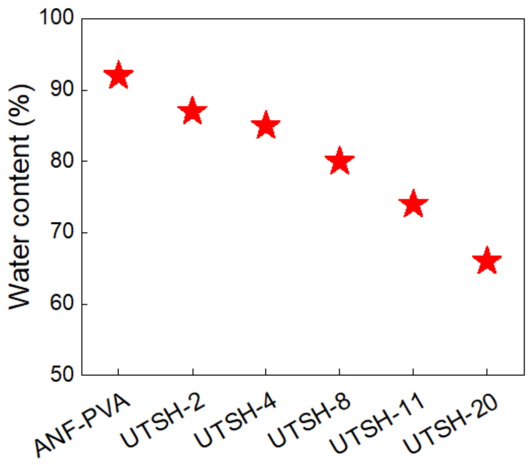

Fig. S9 Water content of UTSHs as a function of TA content. As the TA content increases, the water content of the hydrogel gradually decreases. This may be due to the slight drainage of water from the hydrogel as a result of TA-induced geometric shrinkage. Notably, 74% water content of the UTSH-11 is similar with those of natural tissues, such as skin dermis (~70%)[1].

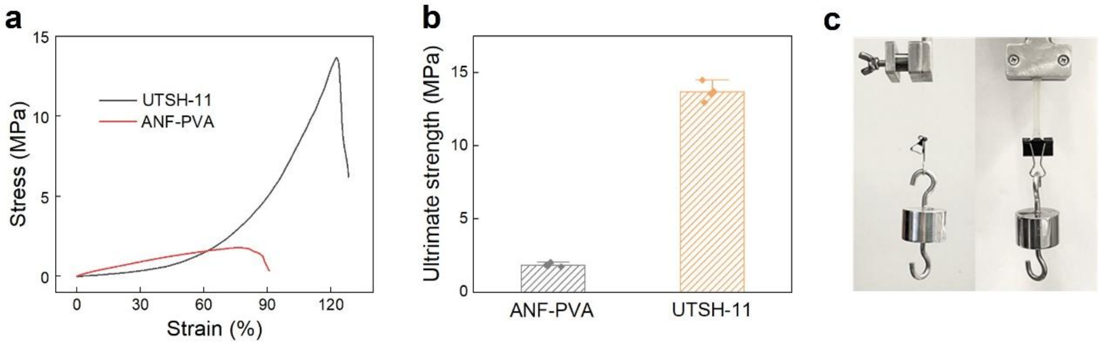

Fig. S10 Comparison of the tensile responses between ANF-PVA and UTSH-11 at the same solid fraction. (**a**) Stress-strain curve. (**b**) Ultimate strength. At the same solid fraction, the TA-reinforced hydrogel demonstrated higher fracture strength and strain, confirming the enhancement of mechanical properties. (**c**) Demonstration of the high strength of UTSH. The samples can bear mechanical loads 5000 times higher than their own weights without fracture or severe distortion, indicating its excellent mechanical performance.

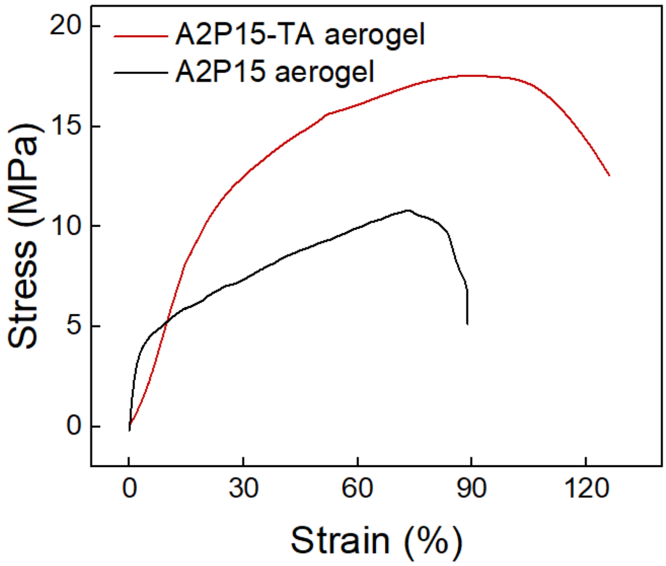

Fig. S11 Comparison of the tensile responses between aerogels derived from UTSH-11 (A2P15-TA) and ANF-PVA hydrogels. The A2P15-TA aerogel exhibits higher ultimate strength and strain, along with a lower initial modulus, resembling the mechanical behavior of hydrogels. This reflects a similar deformation influence in the fibrillar network as described in hydrogels.

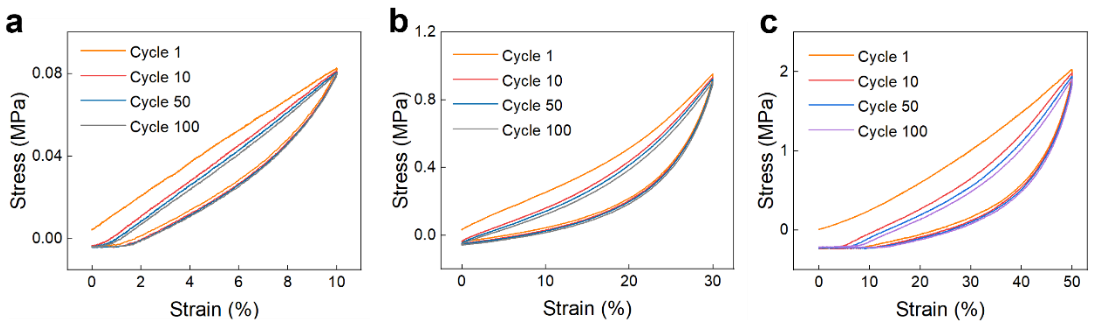

Fig. S12 Cyclic tensile tests of UTSHs under different maximum imposed strains at Cycle 1, Cycle 10, Cycle 50, and Cycle 100. (**a**) Maximum strain of 10%; (**b**) Maximum strain of 30%; (**c**) Maximum strain of 50%.

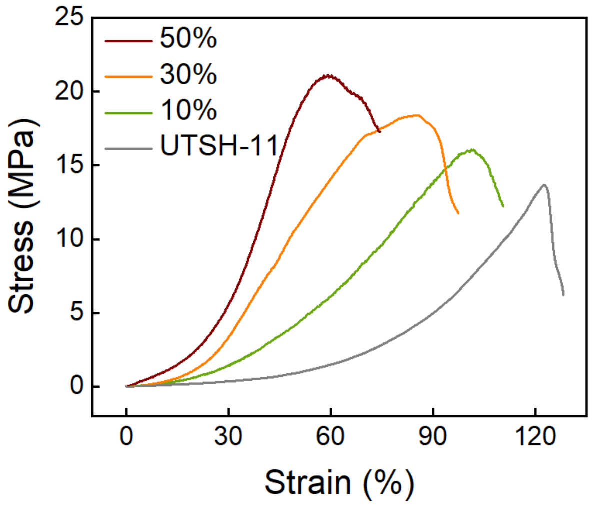

Fig. S13 The tensile responses of UTSHs after 100 times cyclic tensile tests. The unique strain-stiffening characteristic of UTSHs was retained even after cyclic loading.

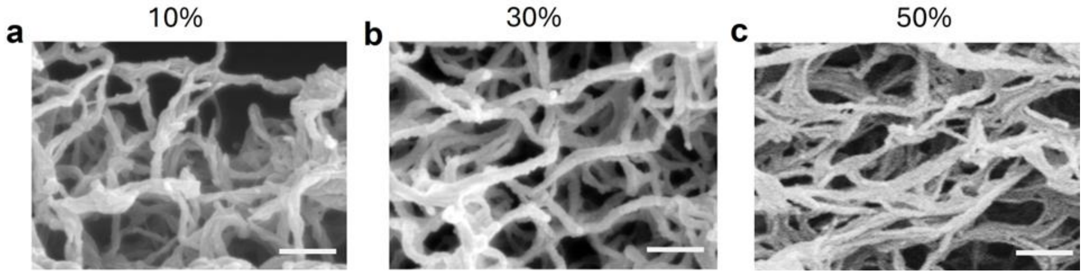

Fig. S14 SEM images of the microfibrillar network in UTSHs after cyclic tensile tests at different maximum strains. (**a**) 10%; (**b**) 30%; (**c**) 50%. Scale bar: 200 nm. These results indicate that the microfibrillar network remains intact.

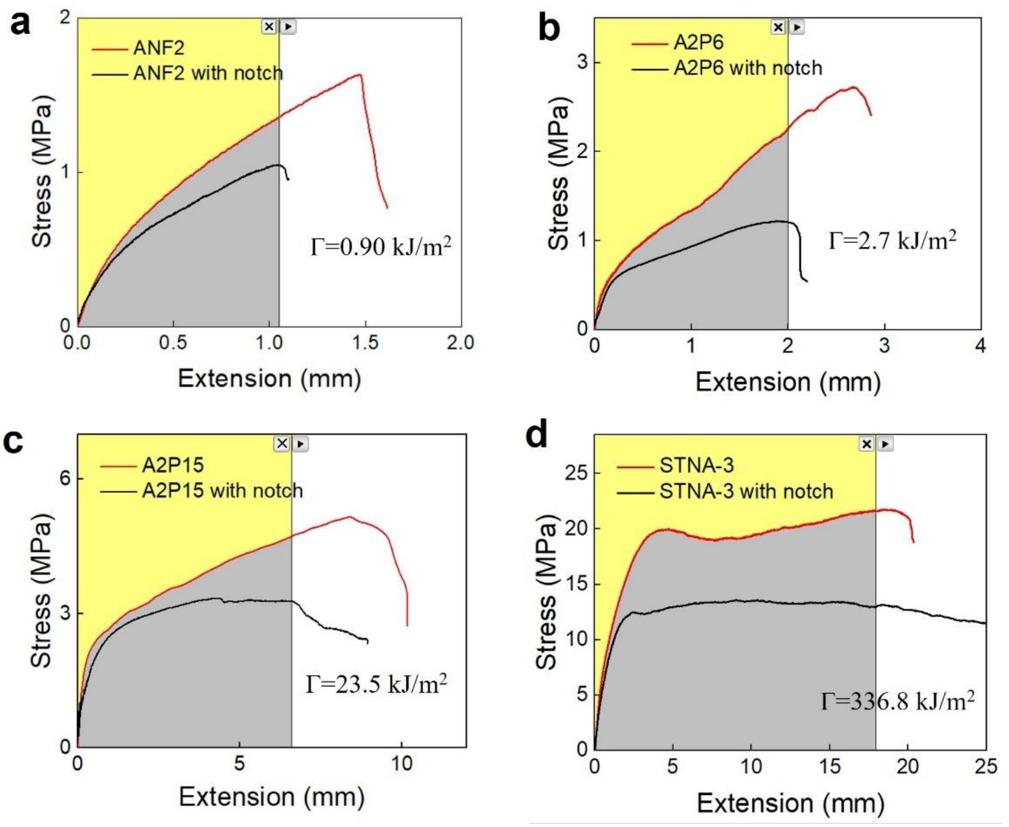

Fig. S15 Calculation of the fracture energy. (**a-d**) Force-extension curves and the corresponding fracture energies of UTSHs with different configurations for A3P15-TA (**a**), UTSH-11(A2P15-TA) (**b**), A1.5P15-TA (**c**) and A1P15-TA (**d**). The fracture energy is given by: $\Gamma = U(\Delta L_c)/A$, where ΔL_c represents the critical extension at which the notch transitions into a propagating crack. U(ΔL_c) denotes the work done on an unnotched sample to achieve an extension of ΔL_c, calculated as the integral of the force-extension curve. A represents the sectional area of the sample.

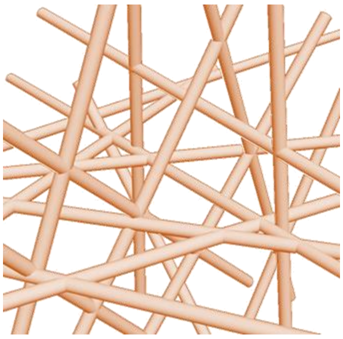

Fig. S16 A schematic of the simulated 3D network. The 3D network is consisting of randomly distributed nanofibers. The diameter, nodal strength, nodal binding energy can be tuned in the modal.

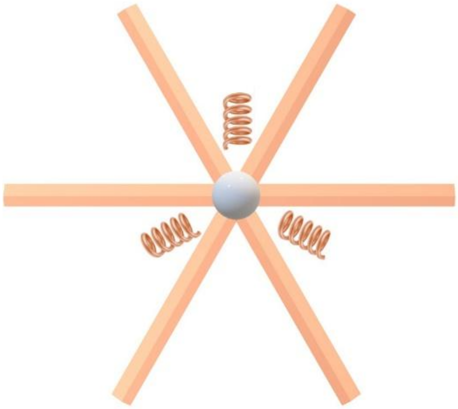

Fig. S17 A schematic illustration featuring linear and rotational springs linking each pair of crosslinked fibrils. Linear and angular springs were added between each pair of fibrils to restrain their relative separation and rotation. For instance, if a welded node contains three crosslinked fibrils, 3 pairs of linear and rotational springs will be introduced to represent the interconnection among them.

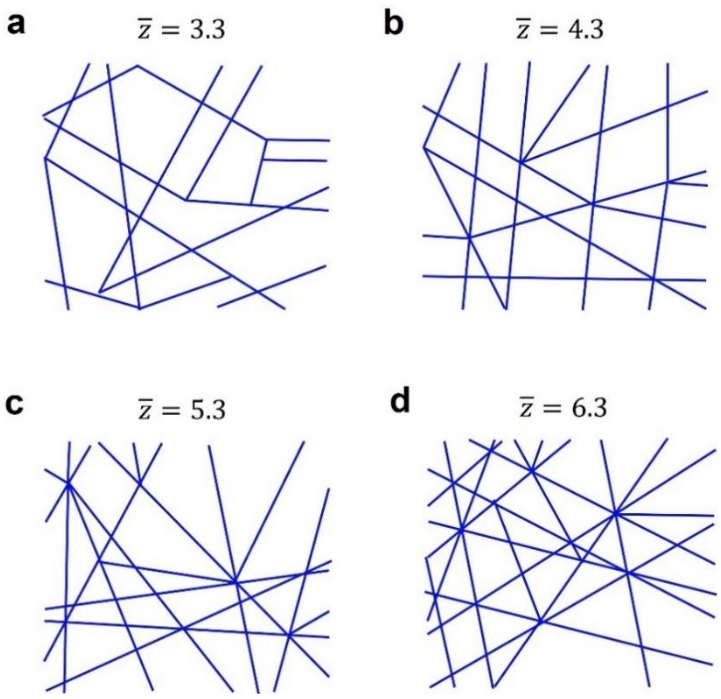

Fig. S18 Representative 3D networks with different average nodal connectivity. The average nodal connectivity ($\bar{\mathbf{z}}$) defined as the average number of fibers connected to each node. (**a-d**) The $\bar{\mathbf{z}}$ values were adjusted to be 3.3 (**a**), 4.3 (**b**), 5.3 (**c**), and 6.3 (**d**), respectively.

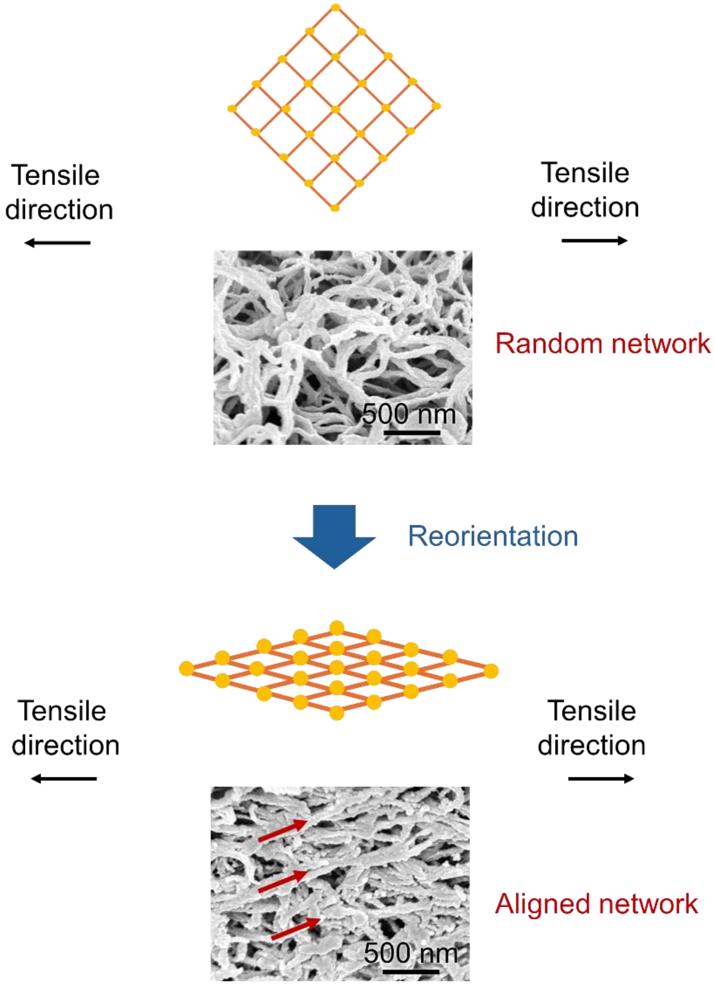

Fig. S19 Reorientation of the Fibrillar Network. Schematics and SEM images illustrate the transformation of a random network (top, before stretching) into an aligned network (bottom, after stretching). During the stretching process, the deformation of the fibrillar network drives its reorientation from a random to an aligned configuration, resulting in an increase of stiffness. Red arrows in the SEM image of the aligned network describe the orientation direction.

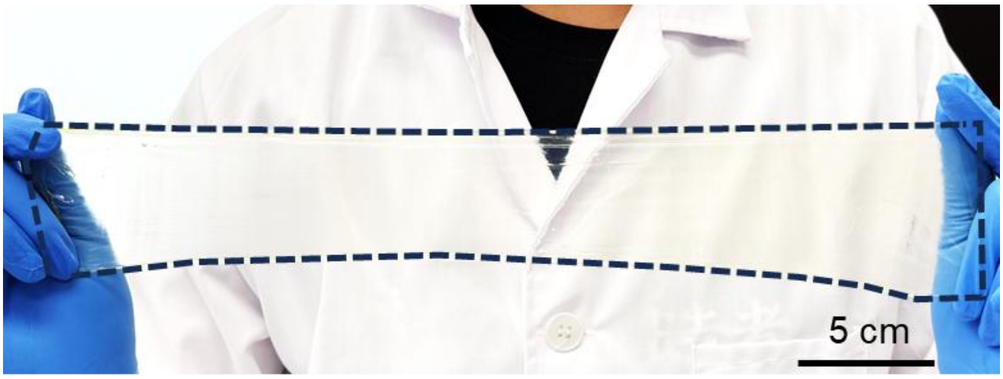

Fig. S20 A photograph of a large-area UTSH film (6 cm × 35 cm × 10 μm). This result confirms the scalability of UTSH for mass production, and the demonstrated size is sufficient to meet the requirements of most wearable and implantable electronic applications. In principle, fabrication of large-area UTSH is feasible; however, in practice, certain limitations remain. Currently, the achievable size of UTSH is primarily restricted by the dimensions of the fabrication equipment, including the coating blade, the supporting substrate, and the available space for maintaining a protective atmosphere. Additionally, when functional components are integrated—such as sensor arrays fabricated via wafer-based microfabrication techniques—the size constraints of the microfabrication equipment and wafer may further limit the functional area.

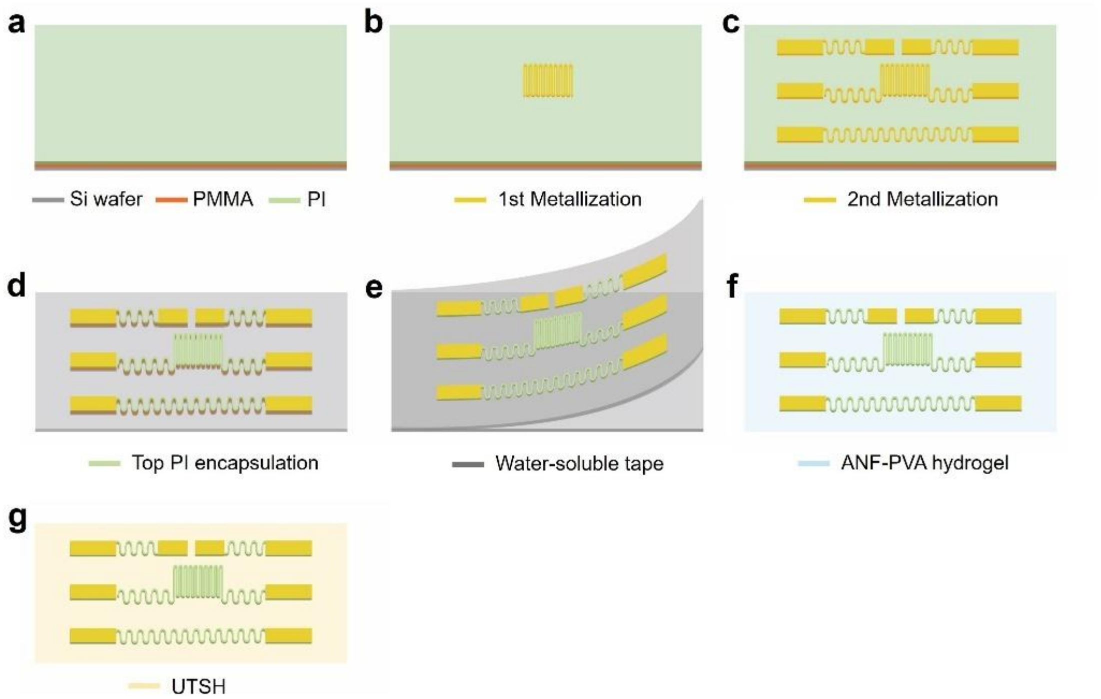

Fig. S21 Schematic illustration of the fabrication and integration of wafer-fabricated sensors with ultrathin ANF–PVA hydrogel membranes. (a) Spin coating of PMMA and polyimide (PI) layers on a 4-inch silicon wafer. (b,c) Thin-film deposition, photolithography, and etching processes for the first and second metallization layers. (d) Deposition of the top PI encapsulation layer, followed by reactive ion etching (RIE) to define device patterns. (e) Transfer of the patterned microelectronic components onto a water-soluble tape after dissolving the PMMA sacrificial layer in acetone, providing temporary mechanical support. Prior to hydrogel integration, the PI surface is treated with oxygen plasma to enhance interfacial adhesion. (f) Heat-assisted blade coating of the ANF–PVA precursor onto the supported microelectronic components under rigid backing, followed by solvent exchange in water to form a uniform, wrinkle-free ANF–PVA hydrogel membrane. The water-soluble tape is gently dissolved during immersion, minimizing mechanical damage. (g) Final formation of the ultrathin ultratough skin-like hydrogel (UTSH) device after tannic-acid (TA) treatment, which reinforces the mechanical robustness and ensures stable integration of the ~10 μm ultrathin hydrogel bioelectronic membrane.

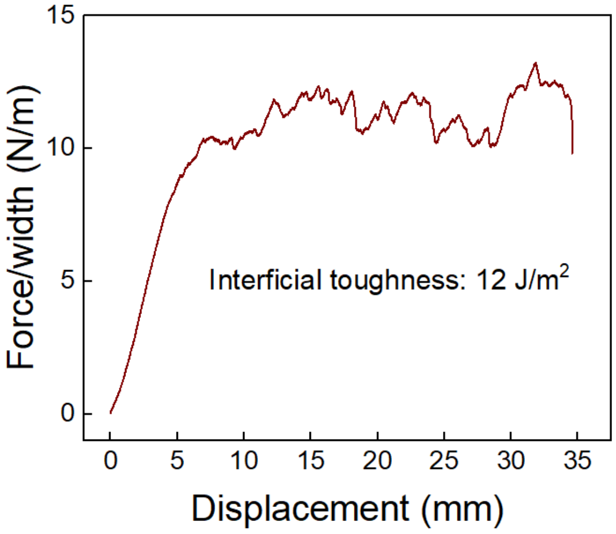

Fig. S22 Characterization of interfacial toughness between transferred components (PI) and UTSH-11. The interfacial toughness between transfer-printed components (PI) and UTSH-11 was estimated to be ~12 J/m^2, indicating the robust interfacial integration between electronic components and the hydrogel membrane.

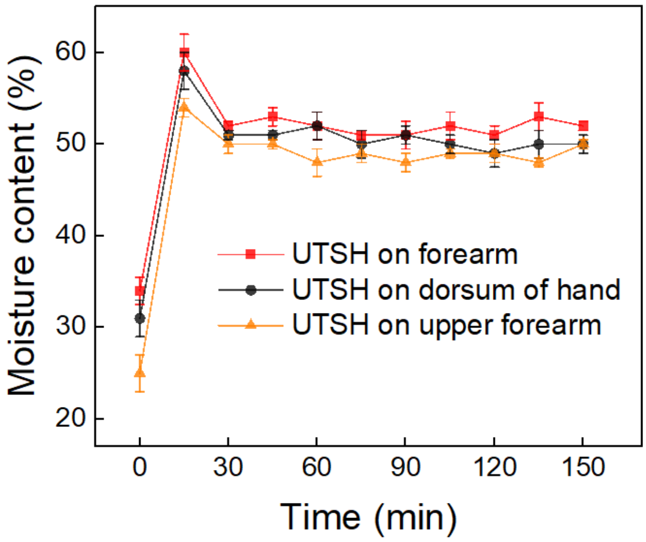

Fig. S23 Measurement of the skin hydration level. The moisture content was measured at three locations on a healthy volunteer by attaching a PAA-coated UTSH film to the skin. Maintaining adequate hydration is crucial for healthy skin, as it enhances the skin's barrier function, elasticity, and overall resilience. Quantitative evaluation using skin impedance demonstrates that the PAA coated UTSH substrate can maintain a high skin hydration level of approximately 50%, indicating the distinct advantages of hydrogel-based epidermal electronics.

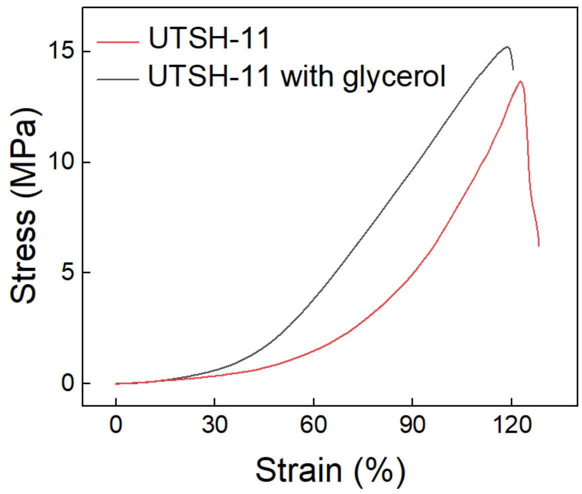

Fig. S24 Comparison of tensile responses of UTSH-11 before and after incorporating glycerol. The glycerol was incorporated by immersing UTSH-11 in a mixed liquid with a weight ratio of glycerol: water= 2:1, the finial water content is 25%. The results showed that the mechanical properties of the hydrogel did not change significantly after the addition of glycerol. This may be because the micromorphology of the network was already established after TA was introduced. As a result, glycerol does not alter the network structure, and therefore has a tiny impact on the mechanical properties.

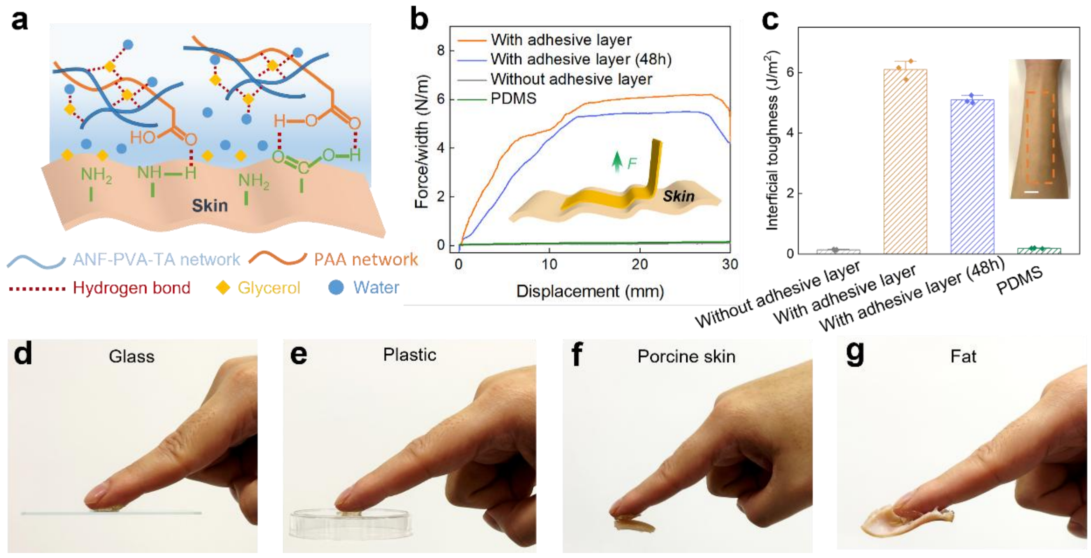

Fig. S25 Characterization of adhesive performance. (**a**) Schematic illustration of the intermolecular interaction between PAA coated UTSH membrane and human skin. *In situ* polymerization of PAA within UTSH can endow the hydrogel with good adhesion capability[2]. The carboxyl groups on the PAA network can form hydrogen bonds and electrostatic interactions with the amino and carboxyl groups on the skin surface, thereby enhancing the adhesion performance[3, 4]. (**b**) Representative curves of the peeling force per width of the hydrogel (Force/Width) versus displacement. (**c**) Comparison of interfacial toughness between a UTSH membrane, a PAA coated UTSH membrane and a PAA coated UTSH membrane after attaching for 48 h and a PDMS film. The inset shows a ~13-μm-thick PAA coated USTH membrane attached to the forearm. The dashed box guides the location of the PAA coated UTSH membrane. The membrane is tightly attached to the forearm with no obvious folds or gaps observed. Scale bar: 4 cm. (**d-g**) Demonstration of the adhesion properties of PAA coated UTSH membrane. In these optical images, one side of PAA coated UTSH membrane is adhered to human skin, while the other side is adhered to glass (**d**), a plastic petri dish (**e**), porcine skin (**f**), and fat (**g**). None of these items detached, indicating the excellent adhesion properties of PAA coated UTSH membrane.

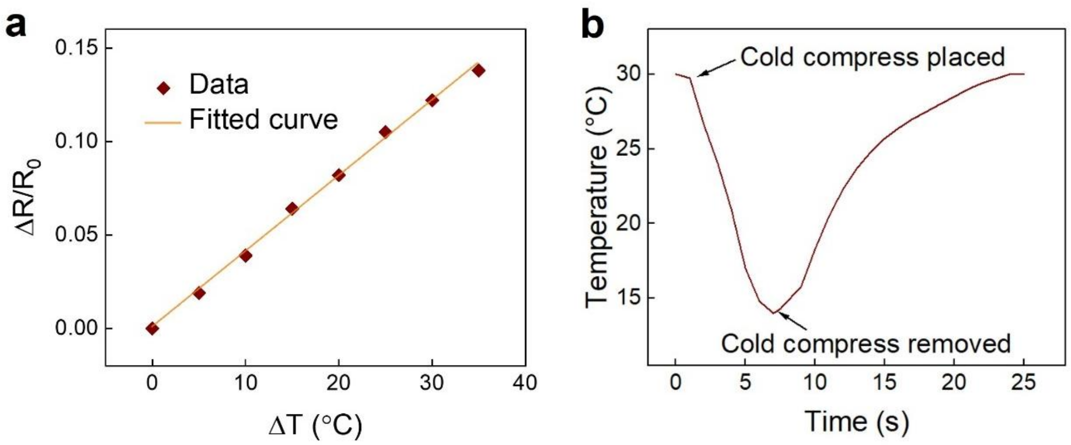

Fig. S26 Characterization of the temperature Sensor. (**a**) Plot showing the resistance change as a function of temperature variation. (**b**) Temperature changes recorded during a cold compression process. The temperature sensor, comprising gold conductive patterns, is integrated onto a PAA-coated UTSH membrane. The resistance change is proportional to the temperature variation, enabling real-time monitoring of temperature during the cold compression process.

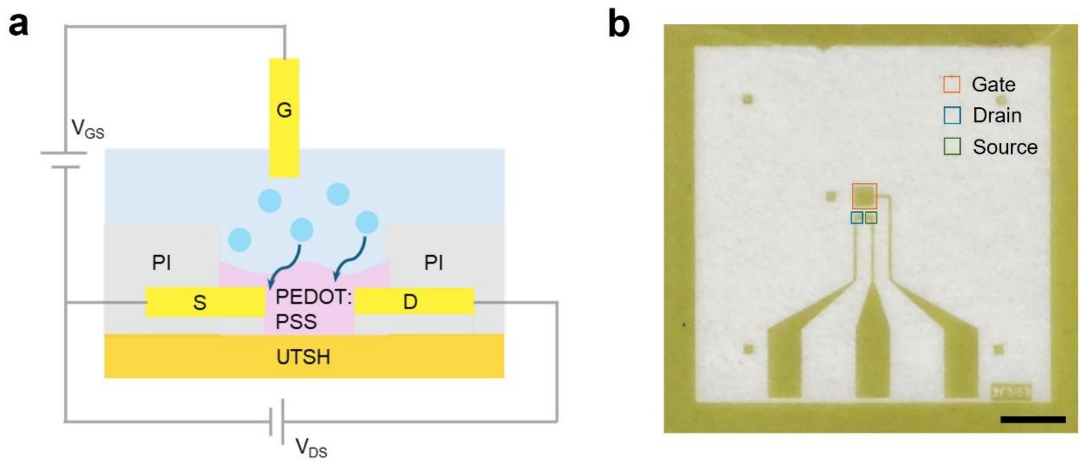

Fig. S27 Fabrication of OECTs on UTSHs. (**a**) Schematic illustration of OECTs integrated onto UTSHs. (**b**) Top-view of the source, drain, and gate electrodes in the OECTs (scale bar: 1 cm). In the OECTs, the source, drain, and gate electrodes were fabricated using gold conductive patterns, while PEDOT:PSS was deposited between the source and drain electrodes, serving as the channel material.

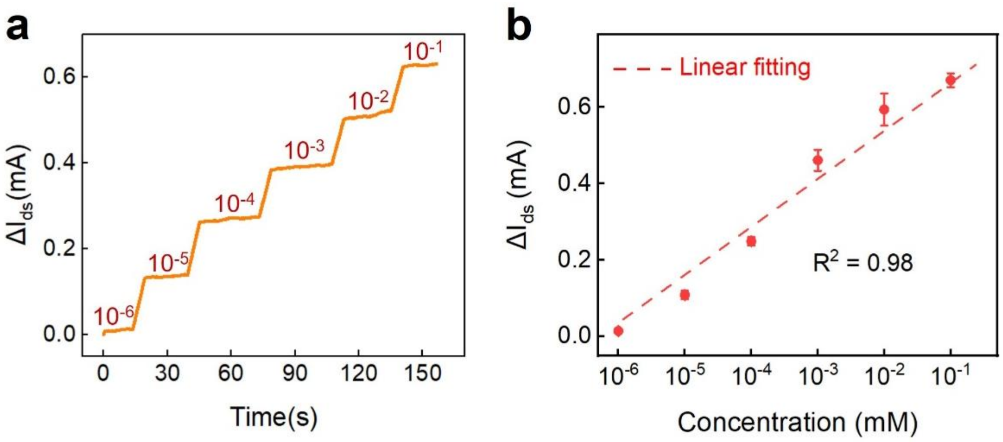

Fig. S28 Measurement of UTSH-based OECT glucose sensor. (**a**) Real-time current response (ΔI_{DS}) of the UTSH-based OECT glucose sensor to glucose concentrations ranging from 10^{-6} to 10^{-1} M, which can meet the requirements of the concentration range (0.06 mM - 0.2 mM) of glucose sensing test in human sweat[5]. (**b**) Linear fitting of the current response versus glucose concentration.

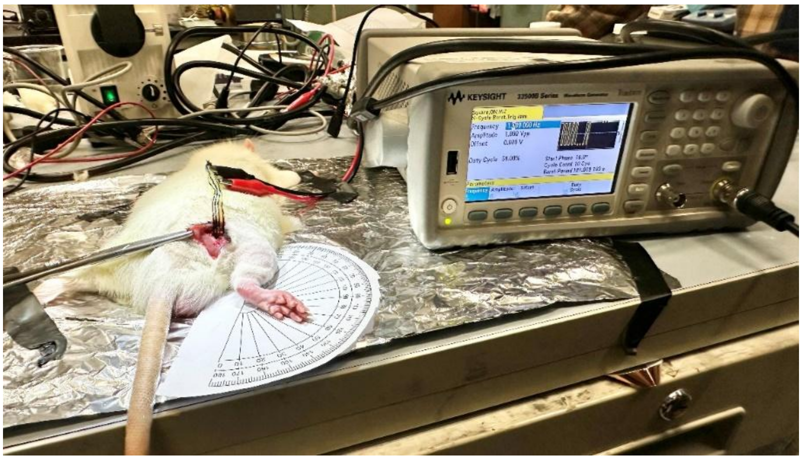

Fig. S29 The setup for the implanted electrical stimulation experiment. The UTSH-PPy electrode is wrapped around the sciatic nerve of a rat and connected to a digital source.

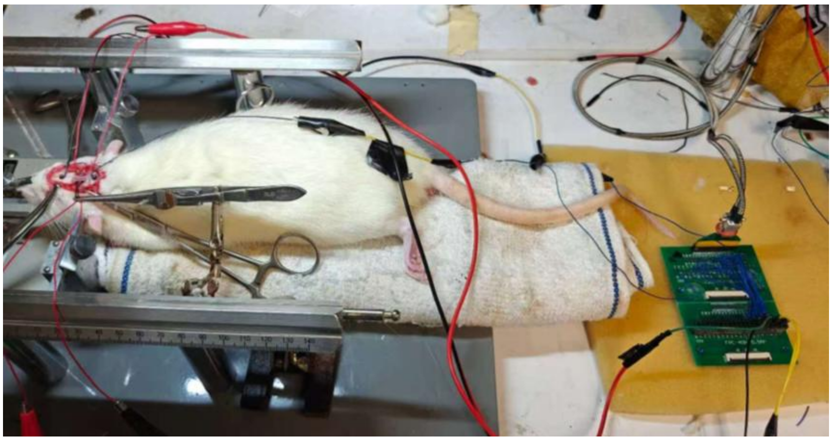

Fig. S30 The setup for the intracranial ECoG recording using the UTSH-PPy electrode. To ensure a more rigorous comparison, the hydrogel and screw electrodes were symmetrically placed in the left and right brain regions of the same mouse.

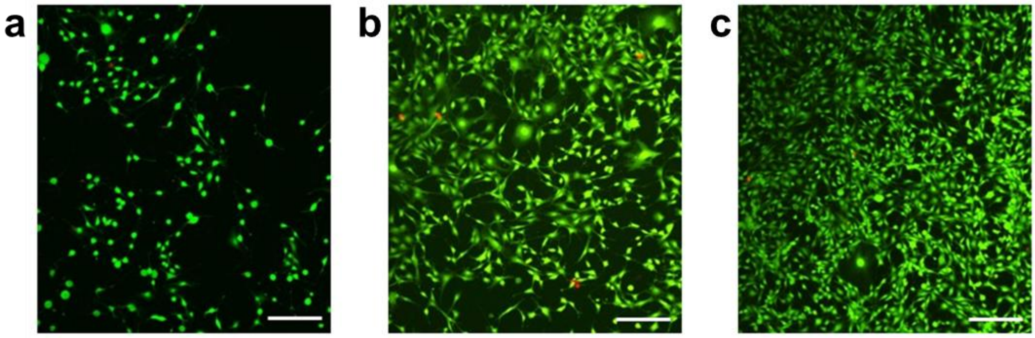

Fig. S31 Cell compatibility of UTSHs. (**a-c**) Live/dead assay of NIH 3T3 fibroblasts cultured on UTSH for 1 (**a**), 3 (**b**), and 5 days (**c**), Scale bar: 500 μm. With the extension of time, the number of cells gradually increased, indicating that UTSHs have excellent biocompatibility.

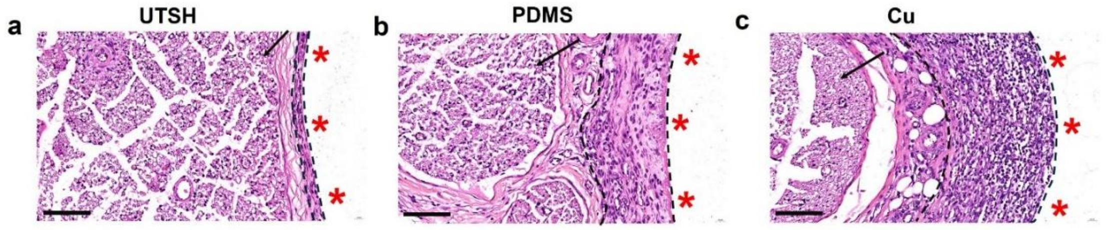

Fig. S32 In vivo biocompatibility. (a-c) Representative histological images of rat sciatic nerves stained with haematoxylin and eosin on day 14 post-implantation of UTSH (**a**), PDMS (**b**) and Cu (**c**). The asterisk denotes the implanted hydrogel bioelectronic interface, while black arrows indicate the sciatic nerve tissue. The region enclosed between two black dashed lines corresponds to the inflammatory infiltration zone. Scale bar: 100 μm.

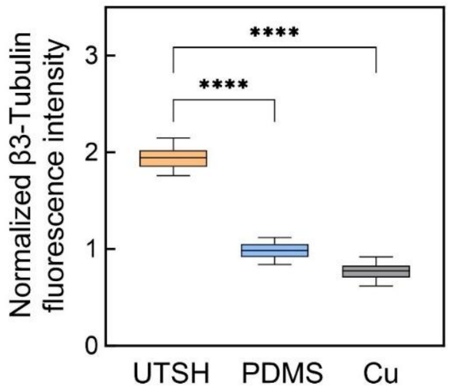

Fig. S33 Normalized fluorescence intensity plots of β3-Tubulin. The higher expression of β3-Tubulin indicates a high level of health after the implantation of UTSH, highlighting its less damage to neural tissues.

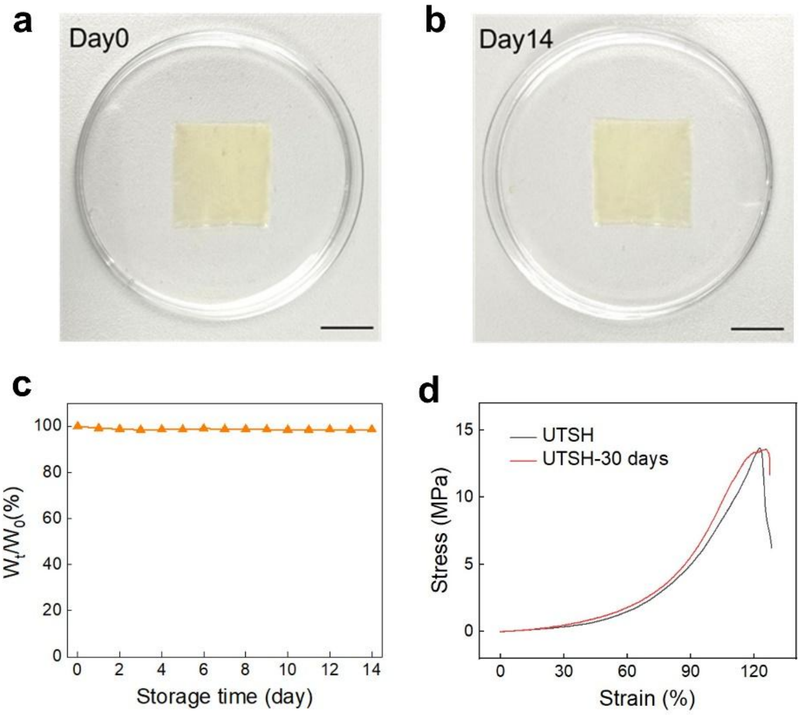

Fig. S34 Evaluation of stability of UTSHs. (a,b) Photograph of UTSHs immersing in PBS solution at the Day0 (just immersed) **(a)** and Day14 (**b**). Scale bar: 2 cm. **(c)** The weight of UTSHs as a function of immersing time. No obvious signs of degradation or swelling were observed, confirming the stability of UTSH. (**d**) Long-term mechanical stability of UTSHs under physiological conditions. Stress–strain curves of pristine UTSHs and UTSHs after 30 days of immersion in saline at 37 °C, showing nearly unchanged mechanical behavior, including modulus and stretchability, indicating excellent long-term mechanical robustness.

Supplementary Tables and Captions

Table S1 Mechanical properties of ANF-PVA and ANF-PVA-TA network at the same fraction

	Ultimate fracture strength (MPa)	Fracture energy (J/m^2)	Modulus (kPa)
ANF-PVA	1.86	1950	370
ANF-PVA-TA	13.65	17800	600

Table S2 Residual plastic deformation after 100 cycles under different maximum imposed strains

Strain	Cycle number	Residual plastic deformation
10%	100 cycles	2.1%
30%	100 cycles	8.6 %
50%	100 cycles	26.8%

Table S3 Mechanical properties of UTSHs in comparison with other hydrogels and natural biological tissues.

Components	Feature	Initial Modulus (E_M, MPa)	Ultimate stress (E_S, MPa)	E_S/E_M	Strain stiffen-ing	BC	Micro-fabrication	Ref
UTSH-11 (A2P15-TA)	Microstructural engineering	0.60	13.65	22.75	√	√	√	This work
A1.5P15-TA	Microstructural engineering	0.56	10.57	18.88	√	√	√	This work
A1P15-TA	Microstructural engineering	0.55	6.7	12.18	√	√	√	This work
Skin	–	0.62	12-16	19.35 -25.8	√	–	–	6
H_2SO_4 treated PEDOT:PSS	Phase separation	888	30	0.03	×	√	√	7
PVA–ceramic platelet	Composite	9.6	7.8	0.81	×	–	×	8
Anisotropic PVA/ cellulose	Microfiber	30	40	1.33	×	√	×	9
Bacterial cellulose-PVA	Composite	877.42	45.06	0.05	×	√	×	10
Alginate	Ionic crosslinking	94-1290	8-57	0.04-0.09	×	√	×	11
Triblock copolymer (PMMA)	Ionic crosslinking	21	0.86	0.04	×	–	×	12
Poly(acrylic acid)–calcium acetate	Phase separation	3.4-131	0.19-17.9	0.06-0.14	×	–	×	13
Polyacrylamide	Dense entanglement	~0.1	0.39	~3.9	×	√	×	14
Polyacrylamide -co-UPyMA	Dense entanglement	0.12	0.2	1.67	×	–	×	15
PVA-polyacrylamide	Microcrystal	5	2.5	0.5	×	√	×	16
Anisotropic PVA	Microcrystal	10	23.5	2.35	×	√	×	17
Anisotropic ANF-PVA	Microfiber	320	72.1	0.23	√	√	√	18
Anisotropic PVA	Microfiber	~4.2	14	3.33	×	√	×	19
Alginate–Polyacrylamide	Double network	0.029	0.156	5.38	×	√	×	20

PEDOT:PSS with d-sorbitol	Phase separation	2.6	~1.2	0.46	×	√	√	21
Alginate-Polyacrylamide -	Double network	0.54	~1.0	1.85	×	√	√	22

Note:

BC: biocompatibility; PEDOT:PSS: poly(3,4-ethylenedioxythiophene)-poly(styrenesulfonate); PMMA: polymethyl methacrylate; UPyMA: 2-(3-(6-methyl-4-oxo-1,4-dihydropyrimidin-2-yl)ureido)ethyl methacrylate; "–": not available.

Table S4 Detailed parameters for the simulation.

Connectivity ($\bar{z}$)	Mean distance (l_c)	Linear spring stiffness (κ_s)	Rotational spring stiffness (κ_r)	Nodal strength (E_s)	Young's modulus (E_m)	Diameter (D)
6.3	0.85 μm	72 μN/ μm	45 nN · μm	10 nN · nm	6 GPa	50 nm
5.3	0.7 μm	72 μN/ μm	45 nN · μm	60 nN · nm	6 GPa	75 nm
4.3	0.64 μm	72 μN/ μm	45 nN · μm	60 nN · nm	6 GPa	75 nm
3.3	0.6 μm	72 μN/ μm	45 nN · μm	60 nN · nm	6 GPa	75 nm

Reference

1. Nakagawa, N., Matsumoto, M. & Sakai, S. In vivo measurement of the water content in the dermis by confocal Raman spectroscopy. *Skin Research and Technology* **16**, 137-141 (2010).
2. Xia, Y. et al. Multifunctional Glycerol–Water Hydrogel for Biomimetic Human Skin with Resistance Memory Function. *ACS Applied Materials & Interfaces* **11**, 21117-21125 (2019).
3. Chen, Z. et al. A conductive and anti-freezing gelatin-PAA-based organic hydrogel (PC-OH) with high adhesion and self-healing activities for wearable electronics. *Chemical Engineering Journal* **492**, 152465 (2024).
4. Yang, G. et al. Adhesive and Hydrophobic Bilayer Hydrogel Enabled On-Skin Biosensors for High-Fidelity Classification of Human Emotion. *Advanced Functional Materials* **32**, 2200457 (2022).
5. Zafar, H., Channa, A., Jeoti, V. & Stojanović, G.M. in Sensors, Vol. 22 (2022).
6. Li, J., Illeperuma, W.R.K., Suo, Z. & Vlassak, J.J. Hybrid Hydrogels with Extremely High Stiffness and Toughness. *ACS Macro Letters* **3**, 520-523 (2014).
7. Yao, B. et al. Ultrastrong, highly conductive and capacitive hydrogel electrode for electron-ion transduction. *Matter* **5**, 4407-4424 (2022).
8. Liu, Q. et al. 3D printable strong and tough composite organo-hydrogels inspired by natural hierarchical composite design principles. *Nature Communications* **15**, 3237 (2024).
9. Wu, L. et al. Natural-Wood-Inspired Ultrastrong Anisotropic Hybrid Hydrogels Targeting Artificial Tendons or Ligaments. *ACS Nano* **17**, 13522-13532 (2023).
10. Xu, H. et al. Ultra-strong mechanical property and force-driven malleability of water-poor hydrogels. *Journal of Colloid and Interface Science* **542**, 281-288 (2019).
11. Ji, D. et al. Superstrong, superstiff, and conductive alginate hydrogels. *Nature Communications* **13**, 3019 (2022).
12. Henderson, K.J., Zhou, T.C., Otim, K.J. & Shull, K.R. Ionically Cross-Linked Triblock Copolymer Hydrogels with High Strength. *Macromolecules* **43**, 6193-6201 (2010).
13. Nonoyama, T. et al. Instant Thermal Switching from Soft Hydrogel to Rigid Plastics Inspired by Thermophile Proteins. **32**, 1905878 (2020).
14. Kim, J., Zhang, G., Shi, M. & Suo, Z. Fracture, fatigue, and friction of polymers in which entanglements greatly outnumber cross-links. *Science* **374**, 212-216 (2021).
15. Xu, J. et al. Low modulus ultra-ductility ionic skin by highly entangled PAM-co-UPyMA hydrogel. *Chemical Engineering Journal* **487**, 150671 (2024).
16. Li, J., Suo, Z. & Vlassak, J.J. Stiff, strong, and tough hydrogels with good chemical stability. *Journal of Materials Chemistry B* **2**, 6708-6713 (2014).
17. Hua, M. et al. Strong tough hydrogels via the synergy of freeze-casting and salting out. *Nature* **590**, 594-599 (2021).
18. Sun, M. et al. Multifunctional tendon-mimetic hydrogels. **9**, eade6973 (2023).
19. Zhu, S. et al. Bioinspired structural hydrogels with highly ordered hierarchical orientations by flow-induced alignment of nanofibrils. *Nature Communications* **15**, 118 (2024).
20. Sun, J.-Y. et al. Highly stretchable and tough hydrogels. *Nature* **489**, 133-136 (2012).

21. Rahman, M.S. et al. Soft, stretchable conductive hydrogels for high-performance electronic implants. **11**, eads4415 (2025).
22. Cheng, S. et al. Ultrathin Hydrogel Films toward Breathable Skin-Integrated Electronics. **35**, 2206793 (2023).
